# Supplementary material for: Effectiveness of a chatbot in improving the mental wellbeing of health workers in Malawi during the COVID-19 pandemic: A randomized, controlled trial
Source: PLoS One. 2024 May 28;19(5):e0303370. doi: 10.1371/journal.pone.0303370 (PMC11132445; doi:10.1371/journal.pone.0303370)
Supplement: S2 File — (PDF) [file pone.0303370.s002.pdf]

S2 – Supplemental Information: Study Protocol

PSYCHOLOGY DEPARTMENT

STUDY TITLE

RANDOMIZED CONTROLLED TRIAL OF THE EFFECTIVENESS OF THE VITALK CHATBOT ON THE MENTAL WELLBEING OF HEALTH WORKERS IN MALAWI

STUDY INVESTIGATOR(S):

ECKHARD KLEINAU

TILINAO LAMBA

EDISTER JAMU

LIMBIKA MALIWICHI

DEMOUBLY KOKOTA

ALEX ZUMAZUMA

SUBMISSION DATE: September 7, 2021

UNIVERSITY OF MALAWI RESEARCH ETHICS COMMITTEE (UNIMAREC)

CHECKLIST FOR ETHICAL REVIEW SUBMISSION

[*To accompany research proposals submitted to the committee for review*]

Note: Before submitting a research proposal to UNIMAREC, an applicant must complete the following checklist by ticking each item in the box and making sure that all the relevant documents corresponding to the ticked boxes are included.

Incomplete submissions will not be processed.

TITLE OF PROPOSAL: RANDOMIZED CONTROLLED TRIAL OF THE EFFECTIVENESS OF THE VITALK CHATBOT ON THE MENTAL WELLBEING OF HEALTH WORKERS IN MALAWI

PRINCIPAL INVESTIGATOR: ECKHARD KLEINAU (COUNTRY LEAD: TILINAO LAMBA)

NAME OF SPONSOR: USAID

AMOUNT OF FUNDING:

I declare that the following items are included in this submission;

1. Covering letter of introduction from the investigator [✓]
2. Three (3) hard copies of the Research Proposal prepared and bound according to UNIMAREC guidelines [✓]
3. A soft copy of the proposal with all the required information as specified below; [✓]

Proposal Title (on cover page) [✓]
Names of Investigators and their Qualifications [✓]
Institution of affiliation (local or international) [✓]
Introduction/ [✓]
Research Problem statement/Justification [✓]
Main and Specific Objectives [✓]
Literature Review [✓]
Description of Methodology/Materials and Methodology/Study design [✓]

- Study sites/locations [✓]
- Study participants [✓]
- Study period [✓]
- Sampling methods [✓]
- Sample size [✓]
- Data collection instruments [✓]
- Data management methods [✓]
- Data analysis method [✓]

Research dissemination strategy [✓]
Ethics

- Risks and strategies for obviating them to enhance protection of rights and welfare of study participants [✓]
- Informed consent form/sheet/assent in English and/or translated into an appropriate local language containing

standard elements of an informed consent form/sheet/assent [✓]

Work plan (including roles of collaborators clearly defined) [✓]
Budget (*that include a **10%** research compliance and capacity building fee when study is approved*) [✓]
Budget justification [✓]
Bibliography [✓]

4. Data collection instruments translated into appropriate local language and referred to in the annex [✓]

5. Letter of approval from foreign ethics committee (for all studying in foreign universities) [ ]

6. Application/Processing fee of US$ 150 or its MKW equivalent [✓]

7. Curriculum vitae (CVs) for all the investigators (in annex) [✓]

SIGNATURE:____________ **NAME (PRINT)** TILINAO LAMBA

DATE: 7TH SEPTEMBER, 2021

DEPARTMENT OF PSYCHOLOGY

Tilinao Lamba
Department of Psychology
Chancellor College
P.O. Box 280,
Zomba, MALAWI

The Chairperson
UNIMAREC
P.O. Box 280
Zomba, MALAWI

Date: 7th September, 2021

Dear Sir/Madam,

SUBJECT: APPLICATION FOR RESEARCH ETHICAL APPROVAL

On behalf of my colleagues, I write to seek an expedited review of the research protocol for ethical approval of our research project, entitled "Randomized Controlled Trial of the Effectiveness of the Vitalk Chatbot on the Mental Wellbeing of Health Workers in Malawi".

The Department of Psychology at the University of Malawi is partnering in this USAID-funded study with Human Resources for Health 2030 (HRH2030) and Chemonics Intl., an international development consulting firm, to investigate the effectiveness of online platform Vitalk, a digital mental health app in improving the mental health indicators of health workers within Blantyre and Lilongwe districts.

Included in this application are the following pieces of documentation:

1. UNIMAREC Checklist
2. $150 UNIMAREC Fee - Bank Slip: exchange rate @ MK888.71:1 USD
3. The research protocol, including:
    a. Research budget
    b. All data collection tools (mental health assessments, questionnaires and FGD guides)
4. CVs of all indicated investigators.

The scheduled end time of the HRH2030 funding for this project is December 2021. Therefore, we are under a tight schedule to conduct this trial and complete all data analysis and reporting by that time. Hence, we appeal to your committee to expedite the review of this research protocol so that we can begin the roll out of this project by Monday 27th September, 2021.

We look forward to your usual timely support in evaluating and approving this application.

Yours faithfully,

Tilinao Lamba
Lecturer – Psychology department
Country Lead – Vitalk RCT project

1. Introduction

This application serves as an expression of interest to conduct research to evaluate the effectiveness of the innovative digital mental health support application "Vitalk" in improving the mental health and resilience of health workers in Malawi.

The details of applicants are presented below, with curriculum vitaes of all researchers appended to this document:

Name	Institution and Position	Academic Qualifications	Areas of Expertise
Eckhard Kleinau	Director of Research and Evaluation, HRH2030/University Research Co. (URC)	**MD** – University of Tübingen<br>**DrPH** – Health Policy & Management, Program Evaluation, Harvard University<br>**MSc** – Health Service Administration, Harvard University<br>**MSc** – Epidemiology, Harvard University	Experimental and quasi-experimental studies, Program evaluation, Implementation research/science
Tilinao Lamba	Lecturer<br>Dept. of Psychology<br>University of Malawi – Chancellor College	**BA** – Psychology (Daystar University, Kenya)<br>**MSc** – Counselling Psychology (Keele University, UK)	Counselling Psychology, psychotherapy, qualitative research (IPA, Content Analysis).
Demoubly Kokota	Lecturer<br>Dept. of Psychology<br>University of Malawi – Chancellor College	**BSOC -** Psychology (University of Malawi)<br>**MPhil -** Mental Health (University of Cape Town, SA)	Public Mental Health, Health psychology, Community psychology, Psychology of Special Populations, Personality Psychology
Limbika Maliwichi	Senior Lecturer,<br>Dept. Of Psychology<br>University of Malawi	**BA –** Psychology & Philosophy, University of Malawi (ZA-MW)<br>**MA** – Clinical Psychology, Sam	Child & Adolescent Mental Health (RCTs)<br>Psychometrics-adaptation of test batteries

		Houston State University (TX-US)<br>**MHS** – Public Mental Health, Johns Hopkins University (MD-US)	Psychotherapy
Edister S. Jamu	Senior Lecturer and Head of Department, Department of Psychology, University of Malawi	**PhD** in Business and Economic Studies (Work and Employment Relations), Leeds University (UK);<br>**MSc** in I/O Psychology and Graduate Diploma in Science (Psychology), University of Western Australia (Perth, Aust.)<br>**BSOC** - University of Malawi	Qualitative research (designing, implementing, analysis, NVIVO); organisation studies, talent management,
Alex Zumazuma	Assistant Lecturer, Department of Mental Health Kamuzu University of Health Sciences (KUHES)	**MBBS**, University of Malawi, College of Medicine<br>**MMed** in Psychiatry, KUHES	General Adult Psychiatry,<br>Child and adolescent mental health services,<br>Psychogeriatrics,<br>Addictions,<br>Psychotherapy,<br>Liaison psychiatry and therapeutics

2. **Background**

Healthcare provision can be stressful even in normal times and maintaining the mental wellbeing of health workers is of utmost importance for optimal and safe patient care (Søvold et al., 2021). The COVID-19 pandemic of 2020/21 has overwhelmed countries' health systems and increased care-related pressure manyfold in the effort of ensuring patient care and staff safety. Added stress of too many life and death decisions, physical exhaustion, lack of protective equipment, and fear of infecting themselves or their families threatens health workers' mental wellbeing daily. Over the last two years, an increasing number of studies report a high proportion of health professionals globally suffering from depression, anxiety, and burnout. A systematic

review of 59 studies by Muller et al. (2020) found that a median of 24% of health workers suffered from anxiety, 21% from depression, and 37% from distress. Nochaiwong et al. (2021) reported slightly higher levels in a meta-analysis from 36 countries – 8.0%for depression; 26.9% for anxiety; 24.1% for post-traumatic stress symptoms; 36.5% for stress; and 50.0% for psychological distress. Recent studies from Sub-Saharan Africa showed similar levels. In a multi-centre cross-sectional study from Ghana, Ofori et al. (2021) found that 21.1%, 27.8% and 8.2% had depression, anxiety and stress, respectively. Research not yet peer reviewed from Ethiopia found a prevalence of depression, anxiety and psychological distress was 20.2%, 21.9% and 15.5% respectively. Data from South Africa suggest much high levels of metal disorders of around 50% among all types of health workers due to work-related stress (Msomi, 2021). While there are no peer-reviewed studies yet from Malawi, initial research using a small sample of nurses and the Coronavirus Anxiety Scale suggests that 26% (n=26) of respondents had COVID-19 related anxiety and 48% (n=49) functional impairment (Chorwe-Sungani, 2021).

Mental disorders not only pose threats to patient safety and health workers' quality of life, but they also come at high economic costs. A World Health Organization (WHO)-led study well before the COVID-19 pandemic found that depression and anxiety disorders cost the global economy US$1 trillion each year. A return on investments between 2.3 and 5.7 to 1 was estimated for scaled up treatment of these mental disorders (Chisholm, et al. 2016). Mental health interventions include basic psychosocial counselling for mild cases, and either basic or more intensive psychosocial treatment plus antidepressant drugs. Over the last decade computer- or internet-based cognitive behavioural therapy (c-CBT or i-CBT) has been tested and implemented as an alternative to in-person treatment of mental health issues. Research has shown that computerized CBT-based self-administered interventions improve depression and anxiety in

adults. A meta-analysis by Grist and Cavanagh (2013) of 49 RCTs revealed a significant medium effect size (g=0.77, 95% CI 0.59-0.95) for computerized CBT (CCBT) for depression and anxiety.

Another meta-analysis by Andrews et al. (2010) of 22 RCTs found an even greater effect size (g=0.88, 95% CI 0.76-0.99). However, in a recent systematic review Christ et al. (2020) found small to medium posttreatment pooled effect sizes regarding depressive symptoms (g=0.51, 95% CI 0.30-0.72) and anxiety symptoms (g=0.44, 95% CI 0.23-0.65) of c-CBT for reducing anxiety and depressive symptoms in adolescents and young adults compared with passive controls. Clinical trials have established that a mobile application can effectively deliver a CBT program for the treatment of depression (Watts et al., 2013), self-management of chronic pain conditions (Kristjánsdóttir et al., 2013) and social anxiety disorder (Dagöö et al., 2014). C-CBT or i-CBT can lower the barrier to seek help and it is especially important where access to psychologists and therapists is low, which is the case in most countries in Sub-Saharan Africa (Bakker et al., 2016).

C-CBT or i-CBT have evolved into interactive chatbots that are driven by artificial intelligence. Chatbots are conversational agents and include the recent entrant Vitalk. Vitalk distinguishes itself that it is available for the public as well as a version adapted for health professionals. The effectiveness of the public version has been established through a panel study without control group by Daley, Hungerbuehler et al. (2020) in Brazil, who found a large post-intervention effect size of Cohen's d of -0.81 or greater for anxiety, depression and stress. The version for health workers has been pilot tested in Malawi, a country where access to mental health therapy is very limited. Statistics on the mental health force in Malawi show that the country has only 0.02 psychologists, 0.01 psychiatrists and 0.04 occupational therapists per 100,000 populations (WHO, 2018). Only recently has the country successfully trained three psychiatrists.

This situation is worsened by the frequent deployment of mental health professionals especially psychiatric nurses to other duties such as maternity services (Kauye, 2008).

To date, there have been few studies for establishing the effectiveness of chatbots and even fewer RCTs. Most of these trials were based on small samples of 70 participants or less, had a short duration of 2-4 weeks, suffered from serious biases, and fell short of establishing that chatbots lead to improved mental health outcomes. All trials were conducted in high-income countries, none in low resource settings (Abd-Alrazaq et al., 2020). No RCTs have been conducted to assess the effectiveness of chatbots in improving the mental health status of health workers specifically (Bakker et al., 2016).

The proposed RCT addresses this evidence gap about the use and effectiveness of chatbots within the health workforce. Building on the earlier pilot test, a RCT will be conducted in Malawi to explore whether a chatbot, Vitalk, is an acceptable source of advice and counselling for health workers to effectively cope with work-related depression, anxiety and burnout and how resilience-building behaviours can mitigate poor mental health outcomes.

3. Research Objectives

Main research objective

The main objective of this study is to test the *null hypothesis that the use of a chatbot such as Vitalk over a period of eight weeks does not result in different mental health outcomes in a treatment and control group* of health workers in Malawi. The null hypothesis will be tested in a two-arm RCT comparing the pre-treatment to post treatment scores using standardized scales for depression, anxiety, resilience, and burnout.

Specific research objectives

In addition to testing the null hypothesis, this study intends to achieve the following specific objectives:

1. To evaluate whether the levels of engagement and continuity of use of Vitalk are adequate to meet the need for mental health support (rate of adoption and continuity).

2. To explore whether the frequency and duration of interaction with Vitalk are significant predictors of mental health outcomes (stickiness/intensity of use).

3. To establish whether the use of Vitalk significantly reduces the number and duration of sub-clinical and clinical episodes of depression, anxiety, resilience, and burnout over the study period (effectiveness).

4. To discover which features of Vitalk are liked or disliked; what improvements are suggested by users; and whether user experience drive stickiness (user experience).

Specific research questions

The specific research questions of this study are as follows:

1. Are the levels of engagement and continuity of use of Vitalk adequate to meet the need for mental health support (rate of adoption and continuity)?

2. Are the frequency and duration of interaction with Vitalk statistically significant predictors of mental health outcomes (stickiness/intensity of use)?

3. Does the use of Vitalk significantly reduce the number and duration of sub-clinical and clinical episodes of depression, anxiety, resilience, and burnout over the study period (effectiveness)?

4. Which features of Vitalk are liked or disliked; what improvements are suggested by users; and does user experience drive stickiness (user experience)?

4. <u>Research Methodology</u>

4.1. Study Design, Randomization, And Blinding

This study is guided by the conceptual framework shown in Figure 1 below. The study is a two-arm, parallel randomized controlled trial with a pre-treatment assessment, 8-week intervention period and mid-study assessments at 4-5 weeks and an end-of-study assessment at 9 weeks[1]. Interested study participants will be randomly assigned to either the treatment or control group at the time of registration. Vitalk, the developer of the app, will create a study web portal where study participants register, give their consent, are assigned to one of the study arms, are given a unique study identification number, and enter their demographic information. Depending on the random assignment, participants will be taken to either the treatment app, Vitalk, or the website for the control group. This is a single blinded design because only the research team will be blinded to the study arm assignments; all participants in both study arms will be told they are participating in '*an online self-help for mental wellbeing*'.

[1] The support from HRH2030 will end December 31, 2021. If additional resources beyond the life of HRH2030 can be mobilized, it is recommended to double the length of the trial to 16 weeks. This would help study the longer-term effects of the chatbot use. One option would be to not cover participants' airtime during this extended period to assess how internet resources would be accessed without such support.

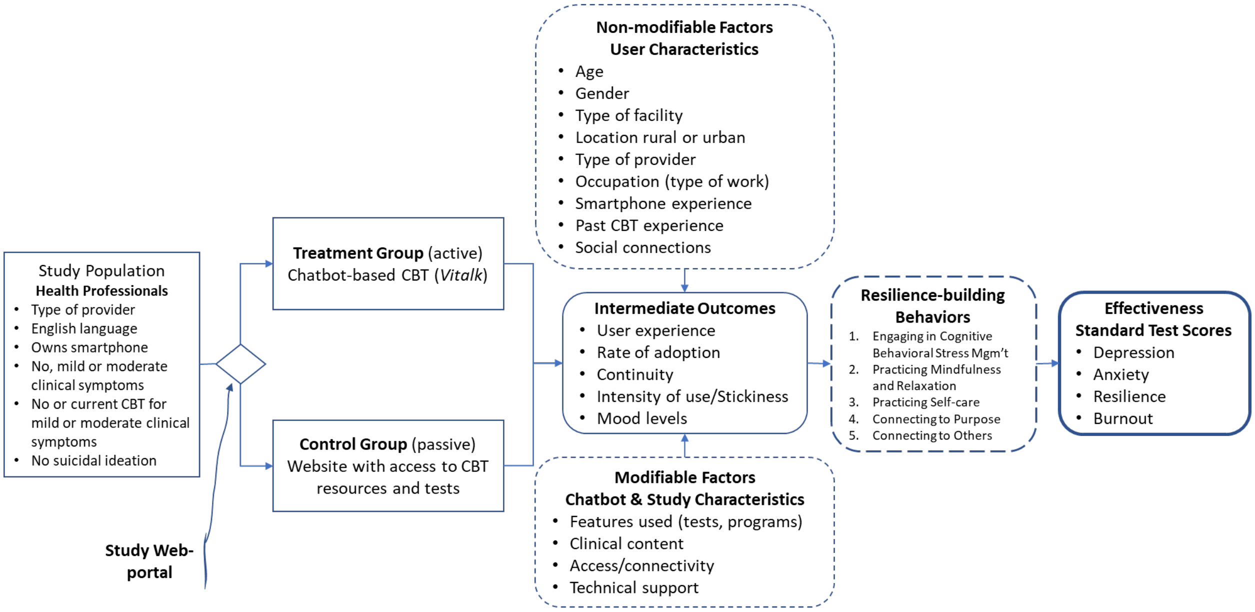

Figure 1. Study framework

Participants will be required to seek approval from their employers to participate in the study, because some activities will occur during working hours, but no information collected during the trial that could identify participants or their facilities of affiliation will be shared with the employers. A trial manager, someone appointed by Vitalk and the Department of Psychology (UNIMA) who is not part of the research team, will provide technical support in case participants encounter difficulties with the study portal or the Vitalk app and be therefore privy to the group assignment, but this information will not be shared with the research team during the study. The study portal and control group website will be optimized for and accessible through smartphones. Vitalk is a smartphone app for Android and iOS.

4.2 Treatment and control group interventions

4.2.1 Vitalk chatbot app (treatment group, *active*)

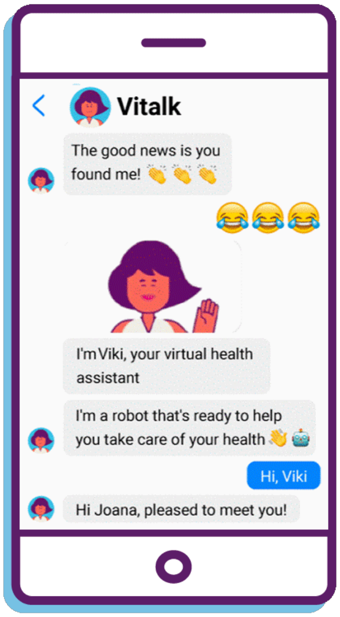

Originating from Brazil, Vitalk is an automated chatbot delivering mental health content to its users using a conversational format, with the aim of improving well-being by reducing stress, anxiety and depression using a preventative approach to mental health (Daley, Hungerbuehler et al., 2020). The Vitalk app uses psychoeducation, cognitive restructuring, behavioral activation, gratitude, and practical exercises (such as breathing, relaxation and meditation) to bring about improvements in users' mental health (ibid). These techniques and strategies are rooted in CBT and Positive Psychology, two commonly used psychotherapy methodologies that have been used widely in various settings and have registered high effectiveness among patients presenting with various psychological challenges (Beck, 2011; Seligman, 1998; Wills, 2009). Only the treatment arm of the study will have access to Vitalk.

4.2.2 Mental health resource website (control group, *passive*)

The control arm of the study will have access to a website with links to mental health resources from WHO and other self-help providers as well as contact information for psychologists and mental health counselors in Malawi. This website consists of a few pages with basic mental health and coping information, links to mental wellbeing resources, mood meter and standardized mental health tests. Its content is static without user interaction beyond clicks to access web-based

mental health resources. Whether or not these resources are used depends entirely on the initiative of control group participants.

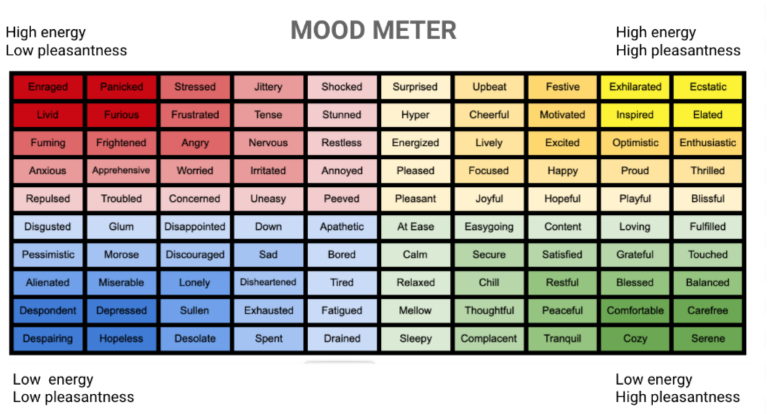

Study sites/locations

The study will be conducted concurrently in both Blantyre and Lilongwe districts, the two biggest cities in Malawi. It will target health workers employed in various health facilities (private, public or mission-managed/religiously affiliated) both within the urban and peri-urban area of both districts.

Study participants

Study participants will be recruited from all primary, secondary and tertiary care facilities within Blantyre and Lilongwe districts.

Inclusion criteria are as follows:

- Type of service provider (cadre)
- English language fluency
- Smartphone ownership
- No or current CBT for mild or moderate mental health disorders
- No suicidal ideation
- Initial mental health test scores below severe levels

The participants of this study will be health workers of the following professional cadres from health facilities:

- Doctors
- Nurses
- Medical Assistants
- Clinical officers
- Laboratory technicians
- Physiotherapy technicians
- Pharmacists

The minimum required educational qualification is a diploma in their respective fields of specialization, in adherence to the Ministry of Health employment criteria for the respective cadres of health workers. However, Medical Assistants who completed 2 years of college will be allowed to participate even though their qualification is a certificate. These selection specifications are put in place based on the general assumption that such participants are competent in the English language as a medium of communication, which will facilitate their interactions with the Vitalk App and the mental health resource website since all the interactions on these platforms are in English. Additionally, study participants are expected to own a smartphone on which they can download and use the Vitalk App and access the mental health resource website, which is an additional reason for limiting participant selection to the above cadres.

Exclusion criteria for this study includes all other hospital- or health-related personnel, such as health surveillance assistants, hospital assistants or laboratory assistants, due to their lower educational qualification requirement for employment. In addition, considering that smart phones are not very widely available within many social circles in Malawi and are considered to many as a luxury, this research assumes that cadres listed above have a higher chance of owning a smart phone due to their higher grade of pay than those of lower employment cadres.

Lastly, a clinical exclusion criteria will apply to all health workers currently under treatment for a severe mental disorder and to all study applicants with severe symptoms of depression, anxiety or burnout according to their initial scores from standard mental health tests. These conditions will include suicidal ideation. Those excluded will not have their identity revealed, but they will simply not be able to stay registered for the study. Participant meeting the exclusion criteria will be offered resources for self-help and contacts of mental health counselors.

Study period

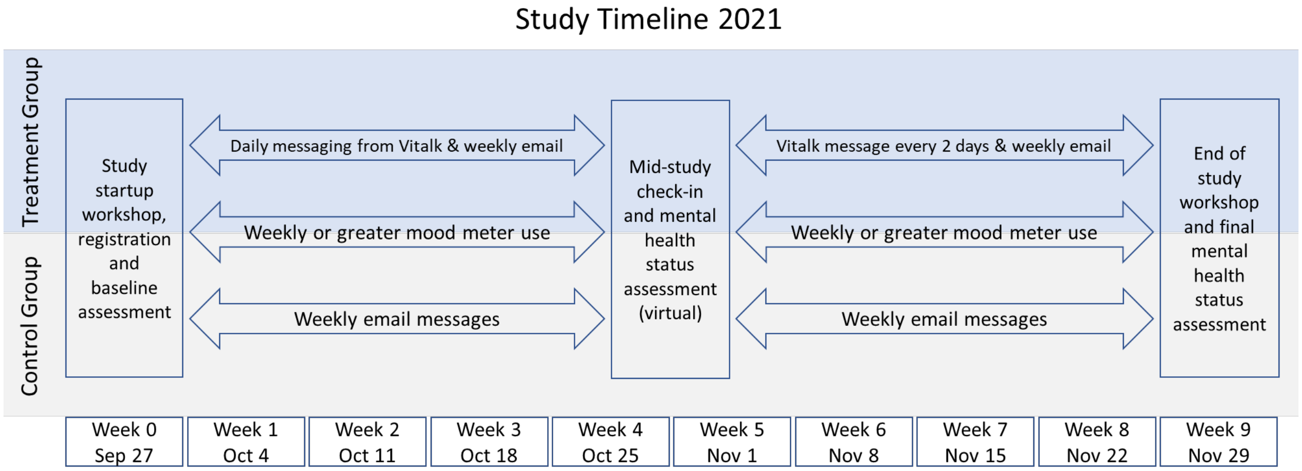

Figure 2. Study process and timeline

This trial is scheduled to span 56 calendar days, from the week beginning on the 4th of October 2021 until the 26th of November 2021. The entire process and timeline are shown in Figure 2. Study participants in both the treatment and control groups will begin their participation in this project by attending a half-day workshop prior to the start of the trial before October 4, which shall serve a dual purpose. Firstly, during this workshop participants shall receive a presentation about general tenets of mental health, how mental health affects work performance, the impact of one's mental health upon their work, and a description of the four specific areas of mental health that

will be assessed: namely anxiety, depression, resilience, and burnout. Secondly, participants will be provided with an overview over the study of '*an online self-help for mental wellbeing*' and the eligibility criteria, but NO specifics about interventions in the treatment or control groups will be mentioned. Only information pertaining to both groups will be discussed.

At the workshop, participants will have the option to join the trial or to leave. Those opting to join will be directed to the study web-portal, provide their consent, complete a pre-screening to establish eligibility, and register for the trial. All participants eligible at this stage shall then complete the initial mental health assessment to assess the initial scores for depression, anxiety, resilience, and burnout. Anybody scoring at the severe level for any of the four scales or showing signs of suicidal ideation will be excluded from the trial and be provided with contacts to mental health professionals in Malawi and Vitalk-affiliated psychologists. All participants passing these final eligibility criteria shall be admitted to the trial and familiarized with links to mental wellbeing resources on the internet and contacts with local psychologists and mental health counselors.

To achieve some degree of participant blinding to the intervention NO information about Vitalk or specifics about interventions in the treatment and control groups will be shared with workshop participants. Participants who completed registration will NOT be informed of their admission into the trial during the workshop; this will happen through an email after the workshop that provides the links to either Vitalk for the treatment group or the website for the control group or informs those not eligible for the trial. The trial manager will monitor whether the desired sample size is achieved; if it is not, additional recruitment efforts and workshops may be necessary.

Participants in the treatment group will receive daily messages and counseling for the first four weeks of the trial to encourage engagement with Viki, the Vitalk virtual counselor. This will

change to every other day during weeks five though eight. Participants in the treatment and control group will receive weekly emails throughout the trial to encourage use of web mental health resources. Both groups will be asked to use the mood meter at least once a week to monitor mood changes. Those self-identifying with extreme negative emotions will be offered to take a relevant standard test again and encouraged to seek professional help.

All participants will be asked to take the standard mental health tests again in week four or five of the trial. This will be done virtually though email to all trial participants as a mid-trial check-in. After the end of the 56-day period, in week nine, both the treatment and control groups will be invited to attend a concluding half-day workshop, during which they shall again be evaluated on their general mental health using the previously completed mental health assessment tools. They shall then complete an anonymous online questionnaire on their experiences of interacting with the app or accessing the referred mental health links. Some participants will be invited to take part in focus group discussions (FGD) aimed at gaining detailed feedback about their experiences of using the two different interventions and the Vitalk app specifically (treatment group participants only for the latter). A mix of in-person and anonymous virtual FGDs are planned given the sensitive nature of mental health. A virtual FGD would be done without video and participants would only be identified by number not name. The use of two FGD formats, in-person and virtual, allows the comparison of how responses may differ between these two approaches. This study concludes with the workshop and FGDs in the 9th-week.

Sampling method

Study participants will be recruited from all primary, secondary and tertiary care facilities within Blantyre district on a first come-first served basis. While this is a convenience sample based

on self-selection, random assignment to treatment and control groups will counteract biases inherent to this approach. All participants are volunteers and agree to be assigned to either the treatment or the control group.

The required study population size for paired tests of correlated means was calculated using STATA 17 and is based on the following:

- Pre-post-treatment difference of 1.5 points on standard test scales
- Standard deviation of the difference = 6
- Power 80%
- Significance level 5%

This results in a minimum of 128 participants per study arm. Given the continuity experience – the proportion of participants completing the pre- and post-treatment assessments for depression, anxiety and stress – of 20-45% reported by Daley, Hungerbuehler et al. (2020), we assume that the dropout rate will be as high as 75% for at least one of the standard mental health assessments. This means that a sample of 512 participants will be required per study arm to yield an effective post intervention sample of 128. Furthermore, if 20% of potential study participants will not meet inclusion criteria or drop out for other reasons, about 640 people will need to be recruited initially per study arm. Therefore, the total number of participants for this study is 1280.

Data collection instruments

This study will collect data on intermediate outcomes related to Vitalk use, resilience-related health behaviors and final outcomes of treatment effectiveness measure as mental health status through standardized tests. In addition, data on independent determinants that potentially influence Vitalk use, resilience-building behaviors and mental health outcomes will be collected.

Resilience-building behaviors can be determinants of mental health outcomes and will be measured as the frequency of five specific behaviors. As shown in Figure 1, these independent determinants fall into two groups: non-modifiable factors related to participant characteristics and modifiable factors related to the Vitalk app and study characteristics. While self-monitoring, knowledge and skills for mental health and coping mechanisms could be among modifiable determinants, the burden of data collection will be too high and outweigh any benefits of obtaining this information, because it would impact response rate negatively.

This study shall utilize the following standardized tests of various mental health indicators, in order to measure the effectiveness of the various mental health platforms that the treatment and control groups shall use:

Generalized Anxiety Disorder (GAD-7)

The GAD-7 is a 7-item self-report scale used to assess anxiety symptoms over the past 2 weeks (e.g., how often have you been bothered by feeling afraid something awful might happen). Scores range from 0 (not at all) to 3 (nearly every day) with a total of 21. The total scores are divided into four categories: none (0–4), mild (5–9), moderate (10–14) and severe (15+) symptoms. GAD-7 has been utilized effectively in Malawi and LMICs with comparable demographics (Mughal et al., 2020).

The Patient Health Questionnaire (PHQ-9)

The PHQ-9 is a 9 item self-report scale that evaluates symptoms of depression over the past 2 weeks (e.g., how often have you been bothered by feeling down, depressed, or hopeless). Item response options use a Likert scale ranging from 0 (not at all) to 3 (nearly every day). Total

scores are divided into five categories: none (0–4), mild (5–9), moderate (10–14), moderately severe (15–19) and severe (20+) symptoms. The PHQ-9 has been widely used and validated in Malawi (Udedi et al., 2019).

14-item Resilience Scale (RS–14)

Resilience refers to the ability to withstand or adaptively recover from stressors. Resilience also promotes psychological and physical well-being. Resilience is negatively correlated with symptoms of generalized anxiety and posttraumatic stress and positively correlated with gratitude, optimism, and positive affect. The five characteristics of resilience are meaning and purposeful life, perseverance, equanimity, self-reliance, and existential aloneness (Wagnild, 2009a). Total scores are categorized as very low (14–56), low (57–64), on the low end (65–73), moderate (74–81), moderately high (82–90), and high (91–98). RS-14 has been validated and used in low and middle-income countries (Wagnild, 2009b; Siriwardhana, 2015)

Oldenburg Burnout Inventory (OLBI)

Burnout is linked to relatively high work requirements and limited resource availability for managing them, which leads to negative emotional states. Therefore, the discrepancy between resources and challenges creates a significant negative emotional state. The OLBI has 16 items, eight to describe exhaustion and eight to describe disengagement (Demerouti & Bakker, 2008). The questionnaire includes both straight and reversely worded items in both dimensions. Low, medium or high OLBI-D scores are based on scores above or below 1 standard deviation of the mean (mean= 2.15, SD = 0.52; ≤1.62= low, 1.63 to 2.67= medium, ≥2.68= high). OLBI has been validated and used in low and middle-income countries (Kaggwa et al., 2021).

Stress is not included in these assessments, because past evaluations have shown almost universally high stress levels pre- and post-intervention, which makes it impossible to use it as an outcome if there are no discriminating factors.

Data management method

The study will collect quantitative and qualitative data. All quantitative data will be collected online through the study portal and the Vitalk app. This will avoid any interviewer or researcher bias. Names, email addresses and phone numbers will be stored only during the trial phase. The data will be de-identified or anonymized prior to analysis and any data sharing by deleting all fields containing identifying information per the Safe Harbor Rule of the U.S. Health Insurance Portability and Accountability Act of 1996 (HIPAA) and subsequent guidance published by the Office for Civil Rights (OCR) at the U.S. Department of Health and Human Services (HHS) (HHS, 2008; HHS, 2012). A participant's clinical trial number will be the unique identifier retained, but it will be encrypted with a secret key to create a pseudonym. Only the trial manager will have a copy of the key. All other identifying information will be permanently deleted. All trial data will be securely stored in the cloud and access to de-identified data restricted to the research team. Access will be controlled by the trial manager for each individual researcher.

Qualitative data will be collected partly online and through focus group discussion (FGD). The concluding workshops in week seven after the end of the 56-day pilot period shall utilize an online questionnaire to be completed anonymously and a FGD guide to gain participant feedback on their experiences of using the app. All focus group discussions shall be recorded, along with notes collected during the FGDs. Once collected and transcribed, all data will be kept in locked

cabinets at the investigator's office. The recordings will be deleted after the completion of data analysis and the journal publication of the study is accepted.

Data analysis method

Data analysis will begin after the final workshop is concluded . There will be no interim sharing or analysis of trial data other than monitoring sample size in the two treatment arms and data completeness and appropriateness per data analysis plan for the entire trial data set. Data monitoring will be done by the trial manager.

The data analysis will start with descriptive statistics and bivariable analyses for mental health outcomes, resilience-building behaviors, intermediate outcomes, and user and study characteristics (non-modifiable and modifiable factors). This will include tests for central tendency and distribution.

The difference between treatment and control arms in mental health outcome scores at baseline, mid-study check-in and end-of-study-will be calculated using a linear, mixed-effect model for longitudinal data (e.g., STATA *mixed* or SPSS *MIXED*) applying maximum likelihood estimation, which takes into account any time trends. This method will allow the simultaneous modelling across different time points and estimate the difference in outcomes between the treatment and control groups across the entire study period.

This study will utilize Interpretative Phenomenological Analysis (IPA) as the main method of analysis for the qualitative data that shall be collected during the focus group discussions. Content Analysis will also be utilized to evaluate the participants' experience in relation to pertinent social and psychological theories in order to explain attitudes and stigma behaviors. This process will also help to identify solutions and interventions that can be applied in order to ensure

increased willingness to properly support health workers facing mental health challenges and a reduction in stigma against mental illness.

Risks

Vitalk is not intended to replace a mental health professional or to offer treatment. Users are made aware of this in the informed consent that each participant is required to sign. Study participants are advised to seek additional support if they show a high risk of depression, anxiety, or stress during the study. Where Vitalk identifies a risk issue, the user is sent details of support services, including the national suicide line, and, if the participant elects the option, a follow up conversation with a healthcare professional from Vitalk who is not affiliated with the study is initiated. Participants identified as high risk or with suicidal tendencies will be removed from the study and referred for mental health support. Under no circumstances will the participant's identity be shared with the research team or anybody outside this study.

The Vitalk App, the mental health resource website and all literature and presentations shared with the research participants shall contain contact information of mental health service providers that the research participants may access in the event of needing further psychological support.

We expect minimal risks to study participants due to COVID-19. The risk of exposure that may be encountered during the workshops is significantly mitigated by the fact that the majority of health workers are vaccinated against COVID-19. Regardless of this, both the research team and participants will be required to maintain the recommended amount of social distance, wear face masks and follow local guidelines as stipulated by the ministry of health.

Benefits/Impacts

The primary benefit of involvement in this project is the anticipated mental health and wellbeing of participants. The overall aim of Vitalk is to improve wellbeing by reducing stress, anxiety and depression using a preventative approach to mental health, which the participants shall benefit from. Additionally, the participants will also benefit from the mental health presentations to be conducted at the workshops, in which the members of the Department of Psychology shall discuss various aspects of mental health and how it affects workplace performance and wellness.

Participants will receive an allotment of 10Gb data bundle allowance valued at K15,500 (about $20) with a selected mobile carrier to eliminate any barriers of accessing the internet during the trial. Those not using the preferred provider at the time of the trial will receive a free SIM card. If it is feasible for the mobile service provider to track whether Vitalk or the control group website were accessed, airtime allocations will be contingent on the use of Vitalk or the control group website. In addition, all participants will be reimbursed $10 for their transport and time, as well as the government rate lunch allowance of K4000 during the attendance of the introductory and concluding workshops.

In addition, and following common practice in clinical trials, participants will receive an appreciation allocation of about 5Gb airtime valued at K8,200 (about $10) at the end of the trial, the final workshop, in recognition of their time sacrifice and as appreciation of their contribution to science. This incentive will hopefully encourage participants in the treatment group to continue the use of the Vitalk app and participants in the control group to try out Vitalk.

5. Research dissemination strategy

The results of this study will be published in an international, peer-reviewed journal and a copy will be submitted to UNIMAREC. These results can also be presented at relevant research conferences, whether local or international. The results of this research will also be disseminated through presentations in fora that will inform policy at a national level and health system strengthening approaches supported by development organizations such as USAID, Chemonics and University Research Co. (URC), and academic institutions such as the University of Malawi. The focus will be on strengthening of mental health services refining and expanding the use of virtual, online tools and innovations.

6. Ethics

The study will be conducted with full adherence to ethical standards as expressed in the APA Code of Ethics on psychological research, as well as the Declaration of Helsinki. Before commencement of the study, relevant authorization will be sought from University of Malawi Research Ethics Committee (UNIMAREC). Participation in the study will be voluntary and participants will be informed of this, and that they may withdraw at any time of the study. Participants who agree to participate in the study will be informed clearly what the study is about and how their information will be used. Informed consent will be obtained online upon registration at the study web-portal. All forms will be printed and kept in a locked cabinet at the Department of Psychology, UNIMA.

7. Workplan

KEY TASKS	Lead Responsible	Aug 9-13	Aug 16-20	Aug23-27	Aug 30-31	Sep 1-3	Sep 6-10	Sep 13-17	Sep 20-24	Sep 27-30	Oct 1-8	Oct 11-15	Oct 18-22	Oct 25-29	Nov 1-5	Nov 8-12	Nov 15-19	Nov 22-26	Nov 29-30	Dec 1-3	Dec 6-10	Dec 13-17	Dec 20-24	Dec 27-30
Draft/submit WP addendum/budget for AOR approval	PMU	X																						
Develop SOW and execute Sub-agreement with Vitalk	PMU		X																					
Develop SOW and execute amendment with URC	PMU		X																					
Develop SOW and execute consultancy agreement with Local Expert	PMU		X																					
Map out steps to develop research protocol for Pilot 2 in Blantyre	Eckhard/ Bruno/ Tilinao		X																					
Develop and submit research proposal to UNIMAREC	Eckhard/ Tilinao			X	X																			
Obtain buy-in from DHO and Blantyre hospitals to reach target number of participants	Tilinao						X	X																
Adapt communications materials: posters, emails	Tilinao						X																	
Identify local venues for workshops	Tilinao				X																			
Develop workshop budget	Tilinao				X																			
Identify local facilitator to provide administrative support to local expert and participants	Tilinao							X																
Identify finance and logistics support	PMU							X																
Translate additional content (15 sessions + 3 audio sessions + mood diary)	Vitalk			X	X																			
Review of additional content	Tilinao					X																		
Upon UNIMAREC Clearance: Launch pilot 2 in Malawi NO LATER THAN OCTOBER 4																								
Communication outreach about Vitalk App: posters, emails	Tilinao								X															
Orientation workshops with all participants	Tilinao, F&A support									X														
Pilot implementation in Blantyre											X	X	X	X	X	X	X	X						
Mid-term check-in workshops with participants VIRTUAL	Tilinao														X									
Mid-term team check-in to monitor and measure process, progress, results	All														X									
Concluding workshops with participants for final check-up	Tilinao																		X					
Data analysis	Eckhard/Tilinao																			X	X			
Adapt How To guide for replication and FAQ document	Vitalk																			X	X			
Draft and disseminate technical reports and other communication products of findings and lessons learned	PMU																			X	X	X		
Draft manuscript for publication	Eckhard/ Tilinao																			X	X	X	X	X

References

Abd-Alrazaq AA, Rababeh A, Alajlani M, Bewick BM, Househ M. (2020) Effectiveness and Safety of Using Chatbots to Improve Mental Health: Systematic Review and Meta-Analysis. J Med Internet Res; 22(7):e16021 URL: http://www.jmir.org/2020/7/e16021/ doi: 10.2196/16021 PMID: 32673216

Andrews G, Cuijpers P, Craske MG, McEvoy P, Titov N. (2010) Computer therapy for the anxiety and depressive disorders is effective, acceptable and practical health care: A meta-analysis. PLoS One;5(10):e13196 [FREE Full text] [doi: 10.1371/journal.pone.0013196] [Medline: 20967242]

Bakker D, Kazantzis N, Rickwood D, Rickard N. (2016) Mental Health Smartphone Apps: Review and Evidence-Based Recommendations for Future Developments. JMIR Mental Health 2016; 3(1):e7. URL: http://mental.jmir.org/2016/1/e7/. doi: 10.2196/mental.4984. PMID: 26932350

Beck J.S. (2011) Cognitive Behaviour Therapy, Second Edition: Basics and Beyond. New York, NY: Guilford Press.

Chisholm D, Sweeny K, Sheehan P, et al. (2016) Scaling-up treatment of depression and anxiety: a global return on investment analysis. The lancet. Psychiatry. May;3(5):415-424. DOI: 10.1016/s2215-0366(16)30024-4. PMID: 27083119.

Chorwe-Sungani, Genesis. (2021) Assessing COVID-19 related anxiety among nurses in Malawi. Research Square. DOI: https://doi.org/10.21203/rs.3.rs-79619/v1

Christ C, Schouten MJE, Blankers M, van Schaik DJF, Beekman ATF, Wisman MA, Stikkelbroek YAJ, Dekker JJM. (2020) Internet and Computer-Based Cognitive Behavioral Therapy for Anxiety and Depression in Adolescents and Young Adults: Systematic Review and Meta-Analysis J Med Internet Res; 22(9):e17831 URL: https://www.jmir.org/2020/9/e17831 doi: 10.2196/17831 PMID: 32673212

Dagöö J, Asplund RP, Bsenko HA, Hjerling S, Holmberg A, Westh S, et al. (2014) Cognitive behavior therapy versus interpersonal psychotherapy for social anxiety disorder delivered via smartphone and computer: A randomized controlled trial. J Anxiety Disord, May; 28(4):410-417. [doi: 10.1016/j.janxdis.2014.02.003] [Medline: 24731441]

Daley, K., Hungerbuehler, I., Cavanagh, K., Claro, H.G., Swinton, P.A. & Kapps, M. (2020) Preliminary Evaluation of the Engagement and Effectiveness of a Mental Health Chatbot. Frontiers in Digital Health. https://doi.org/10.3389/fdgth.2020.576361

Demerouti, E., & Bakker, A.B. (2008) The Oldenburg Burnout Inventory: A good alternative to measure burnout and engagement. In J. R.B. Halbesleben (ed.), Handbook of stress and burnout in health care (65–78). Hauppauge, NY: Nova Science Pub Inc.

Grist R, Cavanagh K. (2013) Computerised Cognitive Behavioural Therapy for Common Mental Health Disorders, What Works, for Whom Under What Circumstances? A Systematic Review and Meta-analysis. J Contemp Psychother, Sep 4; 43(4):243-251. [doi: 10.1007/s10879-013-9243-y]

Hailu Abera Mulatu, Muluken Tesfaye, Esubalew Woldeyes, Tola Bayisa, Henok Fesseha, Rodas Asrat. (2020) The prevalence of common mental disorders among health care professionals during the COVID-19 pandemic at a tertiary Hospital in East Africa. medRxiv 2020.10.29.20222430; doi: https://doi.org/10.1101/2020.10.29.20222430.

HHS. (2008) Coded Private Information or Specimens Use in Research, Guidance. Online https://www.hhs.gov/ohrp/regulations-and-policy/guidance/research-involving-coded-private-information/index.html, accessed 08/25/2021.

HHS. (2012) Guidance regarding methods for deidentification of protected health information in accordance with the Health Insurance Portability and Accountability Act (HIPAA) Privacy Rule. Washington, DC.

Kaggwa MM, Kajjimu J, Sserunkuma J, Najjuka SM, Atim LM, Olum R, et al. (2021) Prevalence of burnout among university students in low- and middle-income countries: A systematic review and meta-analysis. PLoS ONE 16(8): e0256402. https://doi.org/10.1371/journal.pone.0256402

Kauye, F. (2008) Management of mental health services in Malawi. Int Psychiatry.5(2):29–31.

Kristjánsdóttir OB, Fors EA, Eide E, Finset A, Stensrud TL, van DS, et al. (2013) A smartphone-based intervention with diaries and therapist-feedback to reduce catastrophizing and increase functioning in women with chronic widespread pain: randomized controlled trial. J Med Internet Res; 15(1):e5 [doi: 10.2196/jmir.2249] [Medline: 23291270]

Msomi, Nelisiwe. (2021) SA healthcare workers been experiencing burnout long before Covid-19 pandemic.. News24, health 24. Online https://www.news24.com/health24/mental-health/mental-health-in-sa/sa-healthcare-workers-been-experiencing-burnout-long-before-covid-19-pandemic-20210318-2, accessed 08/23/2021.

Mughal, A. Y., Devadas, J., Ardman, E., Levis, B., Go, V. F., & Gaynes, B. N. (2020) A systematic review of validated screening tools for anxiety disorders and PTSD in low to middle income countries. BMC psychiatry, 20(1), 338. https://doi.org/10.1186/s12888-020-02753-3

Muller AE, Hafstad EV, Himmels JPW, Smedslund G, Flottorp S, Stensland SØ, Stroobants S, Van de Velde S, Vist GE. (2020) The mental health impact of the covid-19 pandemic on healthcare workers, and interventions to help them: A rapid systematic review. Psychiatry Res. Nov; 293:113441. doi: 10.1016/j.psychres.2020.113441. Epub 2020 Sep 1. PMID: 32898840; PMCID: PMC7462563.

Nochaiwong, S., Ruengorn, C., Thavorn, K. et al. (2021) Global prevalence of mental health issues among the general population during the coronavirus disease-2019 pandemic: a

systematic review and meta-analysis. Sci Rep 11, 10173. https://doi.org/10.1038/s41598-021-89700-8

Ofori AA, Osarfo J, Agbeno EK, Manu DO, Amoah E. (2021) Psychological impact of COVID-19 on health workers in Ghana: A multicentre, cross-sectional study. SAGE Open Medicine. January. doi:10.1177/20503121211000919

Seligman M.E.P. (1998) Building human strength: psychology's forgotten mission. APA Monitor. 29. doi: 10.1037/e529932010-003

Siriwardhana C, Abas M, Siribaddana S, et al. (2015) Dynamics of resilience in forced migration: a 1-year follow-up study of longitudinal associations with mental health in a conflictaffected, ethnic Muslim population. BMJ Open 2015;5:e006000. doi:10.1136/bmjopen-2014-006000

Søvold Lene E., Naslund John A., Kousoulis Antonis A., Saxena Shekhar, Qoronfleh M. Walid, Grobler Christoffel, Münter Lars. (2021) Prioritizing the Mental Health and Well-Being of Healthcare Workers: An Urgent Global Public Health Priority. Frontiers in Public Health, Volume 9, p 514. https://www.frontiersin.org/article/10.3389/fpubh.2021.679397. DOI=10.3389/fpubh.2021.679397

Udedi, M. (2016) Improving access to mental health services in Malawi. Ministry of Health Policy Brief;26:505–18. Retrieved from https://www.afidep.org/publication/improving-access-to-mental-health-services-in-malawi/

Udedi, M., Muula, A. S., Stewart, R. C., & Pence, B. W. (2019) The validity of the patient health Questionnaire-9 to screen for depression in patients with type-2 diabetes mellitus in non-communicable diseases clinics in Malawi. BMC psychiatry, 19(1), 81. https://doi.org/10.1186/s12888-019-2062-2

Wagnild, G. (2009a) The Resilience Scale user's guide for the US English version of the Resilience Scale and the 14-item Resilience Scale (RS–14). Worden, MT: Resilience Center.

Wagnild G. (2009b) A review of the resilience scale. J Nurs Meas;17:105–13.

Watts S, Mackenzie A, Thomas C, Griskaitis A, Mewton L, Williams A, et al. (2013) CBT for depression: A pilot RCT comparing mobile phone vs. computer. BMC Psychiatry 2013; 13:49 [FREE Full text] [doi: 10.1186/1471-244X-13-49] [Medline: 23391304]

Wills, F. (2009) Beck's Cognitive Therapy; Distinctive Features. London: Routledge. https://doi.org/10.4324/9781315824253

World Health Organization (WHO). (2018) Mental Health Atlas 2017.

APPENDIX A: CONSENT FORMS AND DATA COLLECTION TOOLS

1.1 INFORMED CONSENT FORM

Consent to Participate in Research

Dept. of Psychology, University of Malawi/Human Resources for Health in 2030 (HRH2030), USA

Mobile-phone-based study of *online self-help for mental wellbeing*

Principal Investigator: Dr. Eckhard Kleinau, HRH2030/University Research Co. (URC)

Co-investigators: Tilinao Lamba (Country Lead), Edister Jamu, Demoubly Kokota, Limbika Maliwichi, Dept. of Psychology, University of Malawi; Alex Zumazuma, Queen Elizabeth Central Hospital

You are being asked to take part in a *Mobile-phone-based research study of online self-help for mental wellbeing*. Taking part in research is voluntary. Your decision whether or not to take part will have no effect on the quality of your medical care, academic standing, or your job status. Please ask questions if there is anything about this study you do not understand. You can contact us at Tilinao Lamba, [email] or Eckhard Kleinau, [email].

What is the purpose of this study?
The purpose of this study is to assess whether certain forms of *online self-help for mental wellbeing* are more effective than others. Challenges to mental wellbeing covers depression, anxiety, resilience, and burnout due to work-related or personal pressures.

Are there any benefits from taking part in this study?
You might or might not personally benefit from being in this research study. Through this study we hope to gather information that may help health professionals like you in the future.

What does this study involve?
Your participation in this study may last up to **10 weeks**, beginning on or around September 27, 2021 and ending latest on December 3, 2021. If you elect to participate, you will need a smartphone and access to the internet and study-related websites. Participants will be assigned to different websites at random; you do not have a choice in this matter. The research team will not know to which website you are assigned.

The study team will provide airtime with a mobile carrier contingent on your active participation in the study and, if necessary, a SIM card. During the study period the website will ask you to identify yourself and provide information about your work. You will also be asked to complete four short mental wellbeing tests at the beginning, middle and end of the study. The website will prompt you daily, every other day or weekly and ask you about your mood status, answer your questions and counsel you about any mental health challenges that you might be facing. The website will collect how often you access the study website and the duration of your interaction with different parts of the website. The website does NOT collect other information such as your location or web browsing

history outside the study website. We use the information collected in assessing your levels of mental wellbeing and will share this information with you in real time.

What are the options if you do not want to take part in this study?
Participation is voluntary. You are responsible for seeking approval for participating in this study from your supervisor, if so required; the study personnel will not be responsible for this. If you do not want to take part in the study do not complete this consent form (by clicking Submit below). Once enrolled in the study, you can withdraw fromthe study at any time by following the "leave the study" link on the study website. All identifying information about you will be deleted immediately. All other data related to you will be anonymized and deleted at the conclusion of the trial.

What are the risks involved with being enrolled in this study?
No risks are expected. Should the information you provide indicate that you are feeling overwhelmed by depression, anxiety, resilience, and burnout we will provide contacts in Malawi or online where you can get help. If the signs as acute and severe, we will remove you from the study and refer you for mental health counseling with your consent.

Other important items you should know:

- **Leaving the study:** You may choose to stop your participation in this study at any time; simply follow the "leave the study" link on the study website. Your decision to stop your participation will have no effect on your academic standing or job status.
- **Number of people in this study:** We expect over 1,000 health professionals to enroll in this study.
- **Funding:** This study is funded by the United States Agency for International Development (USAID) through the Human Resources for Health in 2030 (HRH2030) program.
- **Study Implementation:** The study is implemented jointly by the Dept. of Psychology, University of Malawi and the HRH2030 program, USA.
- **Product Development:** If the results of this research are used to develop a product soldfor a profit, you will not share in the profit. You will not receive money from the profits.

How will your privacy be protected?
We value your privacy. For the duration of the study, we will collect your name, mobile number and email address. We will use this information to verify your identity, allocate airtime and any other compensation, and to contact you regularly during the study period with information. At the beginning of the study, you will be assigned a unique identification (ID) number. At the end of the study before data are analyzed we will securely encrypt this identification number and delete all personal identifying information so that the information collected during the study can never be related to you. The analysis will be performed with anonymized data only. We will never keep or share any identifiable information.

The information collected in this study includes your responses to the mental wellbeing test, and certain information about you and your work collected at the time of registration. You will have access to this information. We will store this information on a secure cloud server. The information will be used only for the purpose of this research study as stated earlier in this form and will be deleted when no longer needed. Your identifiable information will not be shared with any third party.

<u>What about the costs of this study?</u>
There is no cost to participate in this study.

<u>Will you be paid to take part in this study?</u>
You will receive a total of 10Gb airtime allowance valued at K15,500 (about $20) with a specific mobile carrier for the time you participate in the study. This airtime allowance is provided contingent on your participation in the study. "*Participation*" means that you access the website on a regular basis as requested. The website automatically uploads the data to our secure server whenever a mobile signal or Wi-Fi is available, so to upload the data simply connect to the internet.

In addition, and following common practice in clinical trials, you will receive an appreciation allocation of about 5Gb airtime valued at K8,200 (about $10) at the end of the trial, the final workshop, in recognition of your time sacrifice and as appreciation of your contribution to science. This incentive will hopefully encourage your continued use of internet resources to help your mental wellbeing.

<u>Whom should you call with questions about this study?</u>
If you have technical problems with our app, email us at[email].

If you have questions or concerns about this study, you can call the research directors for thisstudy Tilinao Lamba, [email], 0885795906, or Eckhard Kleinau, [email].

If you have questions, concerns, complaints, or suggestions about human research, you may contact the Chairperson, UNIMAREC. P.O Box 280. Zomba at [email]

<u>CONSENT</u>

I have read the above information about the *Mobile-phone-based study of online self-help for mental wellbeing*. I agree to participate in this study.

Signature

My email address:

My phone #:

After you click submit we will send you an email message with instructions on how to proceed to the study website and how to participate in the study.

1.2 Data collection instruments

Annex 1.2.1

Screening questionnaire (online)

1. What type of health worker are you? *Please select*
    a. Doctor
    b. Nurse
    c. Clinical officer
    d. Medical Assistant
    e. Physiotherapy technician
    f. Laboratory technician
    g. Pharmacist
    h. Other

2. English language proficiency
    a. Fluent
    b. Some proficiency
    c. None

3. Do you own a smartphone?
    a. Yes
    b. No

4. Are you using TNM as a service provider?
    a. Yes
    b. No

5. Are you currently being counseled or treated for mild or moderate mental health issues?
    a. Yes
    b. No

6. Are you currently being counseled or treated for acute or severe mental health issues?
    a. Yes
    b. No

7. Do you have suicidal thoughts?
    a. Yes
    b. No

Annex 1.2.2

Participant characteristics questionnaire (online)

1. How old are you? *in years*

2. What is your gender:
    a. Male
    b. Female
    c. I prefer not to disclose

3. At what type of health facility do you work?
    a. Tertiary/central hospital
    b. Secondary/district hospital
    c. Primary care facility (clinic, health center, community and rural hospital, maternity unit)

4. What type of facility is it?
    a. Public/government
    b. Private-not-for-profit (for example, CHAM)
    c. Private-for-profit

5. Where is your facility located?
    a. Urban
    b. Peri-urban
    c. Rural

6. In which district is your facility located?
    a. Blantyre
    b. Other ______________________

7. What is the main area in which you work? *Single choice*
    a. Counseling
    b. Dental care
    c. Emergency care
    d. General inpatient care
    e. Intensive care
    f. Laboratory services
    g. Maternity care
    h. Mental health care
    i. Neonatal care
    j. Outpatient care
    k. Pediatric care
    l. Physiotherapy
    m. Radiology services

n. Surgical care
o. Other

8. I use my smartphone for: *check all that apply*

How often (one check per row):	*Daily*	*Several times a week*	*Weekly*	*Less often*	*Never*
a. Browsing the internet	○	○	○	○	○
b. Calling, texting, WhatsApp	○	○	○	○	○
c. Facebook, Twitter	○	○	○	○	○
d. Interactive chat apps	○	○	○	○	○
e. Mobile Money	○	○	○	○	○
f. Playing games	○	○	○	○	○
g. Reading and sending email	○	○	○	○	○
h. Watching videos/movies, listening to music	○	○	○	○	○
i. I don't have a smartphone	☐				

9. Are you currently in therapy or have you had therapy in the past for mental health issues? *Select one*
   a. Currently in therapy
   b. Had past therapy during the last 6 months
   c. Had past therapy during the last 2 years
   d. Had past therapy more than 2 years ago
   e. Never had therapy

10. Social relationships: please answer a few more questions about your relationships with others. Remember, when the term "others" is used, it includes friends, neighbors, or family members. Check the circle that represents your response. *One check mark per row*

	Never *1*	*Once in a while* *2*	*Fairly often* *3*	*Very often* *4*
a. In general, how often do you feel that you lack companionship?	○	○	○	○
b. In general, how often do you feel left out?	○	○	○	○
c. In general, how often do you feel isolated from others?	○	○	○	○

Annex 1.2.3

Data collected by the website for the control group and Vitalk for the treatment group

1. Completed registrations (enrollment)
2. Completed GAD-7 and scores
3. Completed PHQ-9 and scores
4. Completed RS-14 and scores
5. Completed OLBI and scores
6. Frequency of use and results of Mood Meter
7. Completed resilience-building behavior assessment and results
    a. Frequency of Engaging in Cognitive Behavioral Stress Mgm't over last 2 weeks
    b. Frequency of Practicing Mindfulness and Relaxation over last 2 weeks
    c. Frequency of Practicing Self-care over last 2 weeks
    d. Frequency of Connecting to Purpose over last 2 weeks
    e. Frequency of Connecting to Others over last 2 weeks
8. Program selected or clinical content accessed in Vitalk or pages accessed and links followed on control website
9. Frequency, timing and duration of interaction with control website or Vitalk
10. Number and frequency of questions asked
11. Number and frequency of no access to the internet
12. Number and frequency of technical support requested

Annex 1.2.4

Concluding Workshop Questionnaire: Treatment Group

Thank you for joining us for the workshop today and for engaging with us as we have explored health workers' psychosocial wellbeing and interacted with the Vitalk app over the past 56 days. We appreciate your time and participation!

We would like to ask you a few questions about your experience so that we can learn from this process and from your feedback. Your responses will be used to understand how the process went, what went well, and what improvements could be made in the future. All your responses will be anonymous and will not be linked to you now or in the future, and you can stop taking this survey at any time.

We are interested in your thoughts, so if you have anything to share that did not come up in the conversation today or that you want us to know, please feel free to share those thoughts on this form. If you have any questions or concerns, feel free to reach out to today's facilitator.

If you agree to this information and want to continue, please complete the following questions:

1. How many days have you actively used the Vitalk app after the first workshop?
   - 0 days
   - 1 to 7 days
   - 8 to 14 days
   - 15 to 21 days
   - 22 to 28 days
   - 29 to 35 days
   - 36 days or more

2. If you used the app less than 20 days, what was the reason for not using it more often? (You can skip this question if you used the app 20 or more days)

3. How much do you agree with the following statement: "The Vitalk app helped me feel better"?
   - Completely disagree
   - Somewhat disagree
   - Neither agree nor disagree
   - Somewhat agree
   - Completely agree

4. What did you find the most enjoyable and/or most helpful when using the app?

5. What were the things that you did not like or found the least helpful when using the app?

6. What are the main benefits you got from using the Vitalk app?

7. How could we improve the app for the future?

8. How likely are you to recommend the Vitalk app to others?

Not at all likely										Extremely likely
0	1	2	3	4	5	6	7	8	9	10
O	O	O	O	O	O	O	O	O	O	O

9. During these workshops on psychosocial wellbeing of health workers, I felt… (check all that apply):
   - ☐ Energized
   - ☐ Renewed
   - ☐ Bored
   - ☐ Inspired
   - ☐ Overwhelmed
   - ☐ Angry
   - ☐ In agreement with the presenter
   - ☐ In disagreement with the presenter
   - ☐ Other

10. Please explain why you checked the boxes you did.

11. Please provide any other feedback you would like to share on the app or these workshops.

Thank you!

Annex 1.2.5

FOCUS GROUP DISCUSSION GUIDE: TREATMENT GROUP

CLOSING WORKSHOP ON PSYCHOSOCIAL WELLBEING FOR HEALTHWORKERS

DATES:

VENUE:

OBJECTIVE: This qualitative enquiry seeks feedback from the experiences of health workers who had been interacted with the Vitalk App over 56 days as a digital mental health solution aimed at improving on their mental health.

QUESTIONS:

1. What are some of the common mental health challenges that health workers face?
2. What specific/unique mental health challenges have health workers faced due to the COVID-19 pandemic?
3. How do social norms or attitudes affect people's ability to get psychosocial help or counselling?
4. What was your initial expectation of the HRH2030 workshops for the psychosocial wellbeing of health workers?
5. What has your experience of using the Vitalk app been like during the past month?
6. How have you been using the Vitalk app in your everyday life?
7. What did you find enjoyable about the conversations with Viki?
8. What conversations with Viki did you find the most helpful?
9. What conversations with Viki did you find the least helpful?
10. What are the main benefits that you got from using the Vitalk app?
11. Which mental health recommendations did you learn from Viki that you will continue to use from now onwards?
12. How would you explain Vitalk to a person who doesn't know about it?
13. What challenges did you face (if any) in using the app?
14. For the participants who did not use the app consistently, what was the reason for the inconsistency?
15. How do you think the Vitalk app can be improved to make it more engaging and effective?

Annex 1.2.6

Concluding Workshop Questionnaire: Control Group

Thank you for joining us for the workshop today and for engaging with us as we have used online mental health resources to improve health workers' psychosocial wellbeing over the past 56 days. We appreciate your time and participation!

We would like to ask you a few questions about your experience so that we can learn from this process and from your feedback. Your responses will be used to understand how the process went, what went well, and what improvements could be made in the future. All your responses will be anonymous and will not be linked to you now or in the future, and you can stop taking this survey at any time.

We are interested in your thoughts, so if you have anything to share that did not come up in the conversation today or that you want us to know, please feel free to share those thoughts on this form. If you have any questions or concerns, feel free to reach out to today's facilitator.

If you agree to this information and want to continue, please complete the following questions:

1. How many days have you accessed the resources that were provided to you on the mental health support website after the first workshop?

   - ○ 0 days
   - ○ 1 to 7 days
   - ○ 8 to 14 days
   - ○ 15 to 21 days
   - ○ 22 to 28 days
   - ○ 29 to 35 days
   - ○ 36 days or more

2. If you accessed the website for less than 20 days, what was the reason for not using it more often? (You can skip this question if you used the app 20 or more days)

3. How much do you agree with the following statement: "The mental health resource website helped me feel better"?
   - ☐ Completely disagree
   - ☐ Somewhat disagree
   - ☐ Neither agree nor disagree
   - ☐ Somewhat agree
   - ☐ Completely agree

4. What did you find the most enjoyable and/or most helpful about the information provided on the website?

5. What were the things that you did not like or found the least helpful about using the website?

6. What are the main benefits you got from the information provided on the mental health support website?

7. How could we improve the website for the future?

8. How likely are you to recommend the use of the mental health support website to others?

Not at all likely										Extremely likely
0	1	2	3	4	5	6	7	8	9	10
O	O	O	O	O	O	O	O	O	O	O

9. During these workshops on psychosocial wellbeing of health workers, I felt… (check all that apply):
   - ☐ Energized
   - ☐ Renewed
   - ☐ Bored
   - ☐ Inspired
   - ☐ Overwhelmed
   - ☐ Angry
   - ☐ In agreement with the presenter
   - ☐ In disagreement with the presenter
   - ☐ Other

10. Please explain why you checked the boxes you did.

11. Please provide any other feedback you would like to share on the app or these workshops.

Thank you!

Annex 1.2.7

FOCUS GROUP DISCUSSION GUIDE: CONTROL GROUP

CLOSING WORKSHOP ON PSYCHOSOCIAL WELLBEING OF HEALTHWORKERS

DATES:

VENUE:

OBJECTIVE: This qualitative enquiry seeks feedback from the experiences of health workers who had given access to online mental health resources over 56 days as a digital mental health solution aimed at improving on their mental health.

QUESTIONS:

1. What are some of the common mental health challenges that health workers face?
2. What specific/unique mental health challenges have health workers faced due to the COVID-19 pandemic?
3. How do social norms or attitudes affect people's ability to get psychosocial help or counselling?
4. What was your initial expectation of the HRH2030 workshops for the psychosocial wellbeing of health workers?
5. What has your experience of using the mental health resource website been like during the past 56 days?
6. How have you been using the website in your everyday life?
7. What did you find enjoyable about visiting and using the website?
8. What did you find the most helpful about the website?
9. What did you find the least helpful about the website?
10. What are the main benefits that you got from using the mental health resource website?
11. Which mental health recommendations did you learn from the website that you will continue to use from now onwards?
12. How would you explain the mental health resource website to a person who doesn't know about it?
13. What challenges did you face (if any) in using the website?
14. For the participants who did not use the website consistently, what was the reason for the inconsistency?
15. How do you think the mental health resource website can be improved to make it more engaging and effective?
