# Supplementary material for: Effectiveness of a chatbot in improving the mental wellbeing of health workers in Malawi during the COVID-19 pandemic: A randomized, controlled trial
Source: PLoS One. 2024 May 28;19(5):e0303370. doi: 10.1371/journal.pone.0303370 (PMC11132445; doi:10.1371/journal.pone.0303370)
Supplement: S3 File — (PDF) [file pone.0303370.s003.pdf]

Effectiveness of a chatbot in improving the mental wellbeing of health workers in Malawi during the COVID-19 pandemic: A randomized, controlled trial

S3 – Supplemental Information: Questionnaires

Annex 1.2.1 **Screening questionnaire (online)**

1. What type of health worker are you? *Please select*
    a. Doctor
    b. Nurse
    c. Clinical officer
    d. Medical Assistant
    e. Physiotherapy technician
    f. Physiotherapist
    g. Laboratory technician
    h. Pharmacist
    i. Other

2. English language proficiency
    a. Proficient
    b. Some proficiency
    c. None

3. What is the operating system of your smartphone?
    a. Android (*If your phone is not an Apple iPhone then you have an Android-based phone*)
    b. Apple iOS
    c. I do not have a smartphone

4. Do you use your phone to check email?
    a. Yes
    b. No

5. Are you using TNM as a service provider?
    a. Yes
    b. No

6. Are you **currently** being counseled or treated for mild or moderate mental health issues?
    a. Yes
    b. No

7. Have you been counseled or treated for mild or moderate mental health issues in **the past**?
    a. Yes
    b. No

8. Are you **currently** being counseled or treated for acute or severe mental health issues?
    a. Yes
    b. No

9. Have you been counseled or treated for acute or severe mental health issues in **the past**?
    a. Yes
    b. No

Annex 1.2.2 **Participant characteristics questionnaire (online)**

1. How old are you? *in years*

2. What is your gender:
    a. Male
    b. Female
    c. I prefer not to disclose

3. At what type of health facility do you work?
    a. Tertiary/central hospital
    b. Secondary/district hospital
    c. Primary care facility (clinic, health center, community and rural hospital, maternity unit)

4. What type of facility is it?
    a. Public/government
    b. Private-not-for-profit (for example, CHAM)
    c. Private-for-profit

5. Where is your facility located?
    a. Urban
    b. Peri-urban
    c. Rural

6. In which district is your facility located?
    a. Blantyre
    b. Lilongwe
    c. Other ______________________

7. What is the main area in which you work? *Single choice*
    a. Counseling
    b. Dental care
    c. Emergency care
    d. General inpatient care
    e. Intensive care
    f. Laboratory services
    g. Maternity care
    h. Mental health care
    i. Neonatal care
    j. Outpatient care
    k. Pediatric care
    l. Physiotherapy
    m. Radiology services

n. Surgical care
o. Other

8. I use my smartphone for: *check all that apply*

How often (one check per row):	*Daily*	*Several times a week*	*Weekly*	*Less often*	*Never*
a. Browsing the internet	○	○	○	○	○
b. Calling, texting, WhatsApp	○	○	○	○	○
c. Facebook, Twitter	○	○	○	○	○
d. Interactive chat apps	○	○	○	○	○
e. Mobile Money	○	○	○	○	○
f. Playing games	○	○	○	○	○
g. Reading and sending email	○	○	○	○	○
h. Watching videos/movies, listening to music	○	○	○	○	○

9. Are you currently in therapy or have you had therapy in the past for mental health issues? *Select one*
   a. Currently in therapy
   b. Had past therapy during the last 6 months
   c. Had past therapy during the last 2 years
   d. Had past therapy more than 2 years ago
   e. Never had therapy

10. Are you **currently** using a mobile chat app for mental health or wellbeing?
    a. Never
    b. Sometimes
    c. Often

11. Have you used a mobile chat app for mental health or wellbeing **in the past**?
    a. No
    b. Yes

12. Are you **currently** using websites for information about mental health or wellbeing?
    a. Never
    b. Sometimes
    c. Often

13. Have you used websites for information about mental health or wellbeing **in the past**?
    a. No
    b. Yes
14. How has the COVID-19 pandemic affected your workload over the past 12 months?
    My workload
    a. Greatly decreased
    b. Somewhat decreased
    c. Stayed the same
    d. Somewhat increased
    e. Greatly increased

15. How were your daily work hours affected by COVID-19 over the past 12 months?
My work hours

    a. Greatly decreased
    b. Somewhat decreased
    c. Stayed the same
    d. Somewhat increased
    e. Greatly increased

16. How has COVID-19 affected your work-related stress level over the past 12 months?
My stress level

    a. Greatly decreased
    b. Somewhat decreased
    c. Stayed the same
    d. Somewhat increased
    e. Greatly increased

17. Did COVID-19 prevent you from going into work over the past 12 months?

    a. No disruptions at all
    b. Several days over the entire year
    c. Several days every month
    d. Several days every week
    e. I did not work at all

Annex 1.2.3

Concluding Workshop Questionnaire: Treatment Group

Thank you for joining us for the workshop today and for engaging with us as we have explored health workers' psychosocial wellbeing and interacted with the Vitalk app over the past 56 days. We appreciate your time and participation!

We would like to ask you a few questions about your experience so that we can learn from this process and from your feedback. Your responses will be used to understand how the process went, what went well, and what improvements could be made in the future. All your responses will be anonymous and will not be linked to you now or in the future, and you can stop taking this survey at any time.

We are interested in your thoughts, so if you have anything to share that did not come up in the conversation today or that you want us to know, please feel free to share those thoughts on this form. If you have any questions or concerns, feel free to reach out to today's facilitator.

If you agree to this information and want to continue, please complete the following questions:

1. How many days have you actively used the Vitalk app after the first workshop?
    - 0 days
    - 1 to 7 days
    - 8 to 14 days
    - 15 to 21 days
    - 22 to 28 days
    - 29 to 35 days
    - 36 days or more

2. If you used the app less than 20 days, what was the reason for not using it more often? (You can skip this question if you used the app 20 or more days)

3. How much do you agree with the following statement: "The Vitalk app helped me feel better"?
    - Completely disagree
    - Somewhat disagree
    - Neither agree nor disagree
    - Somewhat agree
    - Completely agree

4. What did you find the most enjoyable and/or most helpful when using the app?

5. What were the things that you did not like or found the least helpful when using the app?

6. What are the main benefits you got from using the Vitalk app?

7. How could we improve the app for the future?

8. How likely are you to recommend the Vitalk app to others?

Not at all likely										Extremely likely
0	1	2	3	4	5	6	7	8	9	10
○	○	○	○	○	○	○	○	○	○	○

9. During these workshops on psychosocial wellbeing of health workers, I felt…
   (check all that apply):
   - ☐ Energized
   - ☐ Renewed
   - ☐ Bored
   - ☐ Inspired
   - ☐ Overwhelmed
   - ☐ Angry
   - ☐ In agreement with the presenter
   - ☐ In disagreement with the presenter
   - ☐ Other

10. Please explain why you checked the boxes you did.

11. Please provide any other feedback you would like to share on the app or these workshops.

Thank you!

Annex 1.2.4

FOCUS GROUP DISCUSSION GUIDE: TREATMENT GROUP

CLOSING WORKSHOP ON PSYCHOSOCIAL WELLBEING FOR HEALTHWORKERS

DATES:

VENUE:

OBJECTIVE: This qualitative enquiry seeks feedback from the experiences of health workers who had been interacted with the Vitalk App over 56 days as a digital mental health solution aimed at improving on their mental health.

QUESTIONS:

1. What are some of the common mental health challenges that health workers face?
2. What specific/unique mental health challenges have health workers faced due to the COVID-19 pandemic?
3. How do social norms or attitudes affect people’s ability to get psychosocial help or counselling?
4. What was your initial expectation of the HRH2030 workshops for the psychosocial wellbeing of health workers?
5. What has your experience of using the Vitalk app been like during the past month?
6. How have you been using the Vitalk app in your everyday life?
7. What did you find enjoyable about the conversations with Viki?
8. What conversations with Viki did you find the most helpful?
9. What conversations with Viki did you find the least helpful?
10. What are the main benefits that you got from using the Vitalk app?
11. Which mental health recommendations did you learn from Viki that you will continue to use from now onwards?
12. How would you explain Vitalk to a person who doesn’t know about it?
13. What challenges did you face (if any) in using the app?
14. For the participants who did not use the app consistently, what was the reason for the inconsistency?
15. How do you think the Vitalk app can be improved to make it more engaging and effective?

Annex 1.2.5

Concluding Workshop Questionnaire: Control Group

Thank you for joining us for the workshop today and for engaging with us as we have used online mental health resources to improve health workers' psychosocial wellbeing over the past 56 days. We appreciate your time and participation!

We would like to ask you a few questions about your experience so that we can learn from this process and from your feedback. Your responses will be used to understand how the process went, what went well, and what improvements could be made in the future. All your responses will be anonymous and will not be linked to you now or in the future, and you can stop taking this survey at any time.

We are interested in your thoughts, so if you have anything to share that did not come up in the conversation today or that you want us to know, please feel free to share those thoughts on this form. If you have any questions or concerns, feel free to reach out to today's facilitator.

If you agree to this information and want to continue, please complete the following questions:

1. How many days have you accessed the resources that were provided to you on the mental health support website after the first workshop?

   - ○ 0 days
   - ○ 1 to 7 days
   - ○ 8 to 14 days
   - ○ 15 to 21 days
   - ○ 22 to 28 days
   - ○ 29 to 35 days
   - ○ 36 days or more

2. If you accessed the website for less than 20 days, what was the reason for not using it more often? (You can skip this question if you used the app 20 or more days)

3. How much do you agree with the following statement: "The mental health resource website helped me feel better"?
   - ☐ Completely disagree
   - ☐ Somewhat disagree
   - ☐ Neither agree nor disagree
   - ☐ Somewhat agree
   - ☐ Completely agree

4. What did you find the most enjoyable and/or most helpful about the information provided on the website?

5. What were the things that you did not like or found the least helpful about using the website?

6. What are the main benefits you got from the information provided on the mental health support website?

7. How could we improve the website for the future?

8. How likely are you to recommend the use of the mental health support website to others?

Not at all likely										Extremely likely
0	1	2	3	4	5	6	7	8	9	10
O	O	O	O	O	O	O	O	O	O	O

9. During these workshops on psychosocial wellbeing of health workers, I felt… (check all that apply):
   - ☐ Energized
   - ☐ Renewed
   - ☐ Bored
   - ☐ Inspired
   - ☐ Overwhelmed
   - ☐ Angry
   - ☐ In agreement with the presenter
   - ☐ In disagreement with the presenter
   - ☐ Other

10. Please explain why you checked the boxes you did.

11. Please provide any other feedback you would like to share on the app or these workshops.

Thank you!

Annex 1.2.6

FOCUS GROUP DISCUSSION GUIDE: CONTROL GROUP

CLOSING WORKSHOP ON PSYCHOSOCIAL WELLBEING OF HEALTHWORKERS

DATES:

VENUE:

OBJECTIVE: This qualitative enquiry seeks feedback from the experiences of health workers who had given access to online mental health resources over 56 days as a digital mental health solution aimed at improving on their mental health.

QUESTIONS:

1. What are some of the common mental health challenges that health workers face?
2. What specific/unique mental health challenges have health workers faced due to the COVID-19 pandemic?
3. How do social norms or attitudes affect people's ability to get psychosocial help or counselling?
4. What was your initial expectation of the HRH2030 workshops for the psychosocial wellbeing of health workers?
5. What has your experience of using the mental health resource website been like during the past 56 days?
6. How have you been using the website in your everyday life?
7. What did you find enjoyable about visiting and using the website?
8. What did you find the most helpful about the website?
9. What did you find the least helpful about the website?
10. What are the main benefits that you got from using the mental health resource website?
11. Which mental health recommendations did you learn from the website that you will continue to use from now onwards?
12. How would you explain the mental health resource website to a person who doesn't know about it?
13. What challenges did you face (if any) in using the website?
14. For the participants who did not use the website consistently, what was the reason for the inconsistency?
15. How do you think the mental health resource website can be improved to make it more engaging and effective?

Annex 1.2.7

MENTAL HEALTH ASSESSMENTS

Generalized Anxiety Disorder (GAD-7)
What is anxiety?
Some people experience anxiety as a constant worried or restless feeling. For other people, it shows itself as a concrete fear of something specific, a phobia, and can even cause panic attacks.
Please answer the following questions by telling us how you truly feel

Think about the following questions, considering the last TWO WEEKS

Have you felt nervous, anxious or on edge?	Not at all<br>Several days<br>More than half the days<br>Nearly every day
Have you been unable to stop or control worrying?	Not at all<br>Several days<br>More than half the days<br>Nearly every day
Have you worried too much about many different things?	Not at all<br>Several days<br>More than half the days<br>Nearly every day
Tell me, have you experienced difficulty relaxing?	Not at all<br>Several days<br>More than half the days<br>Nearly every day
Have you been so restless that it's hard to sit still?	Not at all<br>Several days<br>More than half the days<br>Nearly every day
Have you become easily annoyed or irritable?	Not at all<br>Several days<br>More than half the days<br>Nearly every day
Have you felt afraid, as if something awful might happen?	Not at all<br>Several days<br>More than half the days<br>Nearly every day

Scoring

0-4	None = Very low risk
5-9	Mild = Low risk
10-14	Moderate = High risk
15-21	Severe = Very high risk

Patient Health Questionnaire (PHQ-9)
What is depression?
Depression is when you experience discouragement, sadness or indifference which seems to be everlasting, and affects all aspects of your life.
You can also feel a deep tiredness, lack of perspective and pleasure in life in general.
Please answer the following questions by telling us how you truly feel

Think about the last 2 WEEKS...

Have you had little interest or pleasure in doing things?	Not at all<br>Several days<br>More than half the days<br>Nearly every day
Have you felt down, depressed or hopeless?	Not at all<br>Several days<br>More than half the days<br>Nearly every day
Have you had troubling falling or staying asleep, or sleeping too much?	Not at all<br>Several days<br>More than half the days<br>Nearly every day
Have you felt tired or had little energy?	Not at all<br>Several days<br>More than half the days<br>Nearly every day
And have you experienced poor appetite or overeating?	Not at all<br>Several days<br>More than half the days<br>Nearly every day
Have you felt bad about yourself, or that you are a failure or have let yourself or your family down?	Not at all<br>Several days<br>More than half the days<br>Nearly every day
Have you experienced difficulty concentrating on actions such as reading or watching television?	Not at all<br>Several days<br>More than half the days<br>Nearly every day
Have you been moving or speaking so slowly that other people could have noticed? Or have you been so fidgety or restless that you have been moving around a lot more than usual?	Not at all<br>Several days<br>More than half the days<br>Nearly every day

Have you thought that it would be better if you were dead, or have you thought about harming yourself somehow?	Not at all<br>Several days<br>More than half the days<br>Nearly every day

Scoring

0-4	None = Very low risk
5-9	Mild = Low risk
10-19	Moderate = High risk
20-27	Severe = Very high risk

Oldenburg Burnout Inventory (OLBI)
What is Burnout?
Burnout is the highest state of occupational stress.
A person's work conditions cause them to reach their emotional and physical limits.
Answer these statements by indicating your degree of agreement or disagreement.
Please read each question carefully because each is worded differently.

"I always find new and interesting aspects in my work."	Strongly disagree<br>Disagree<br>Agree<br>Strongly agree
"There are days when I feel tired before I arrive at work."	Strongly disagree<br>Disagree<br>Agree<br>Strongly agree
"It happens more and more often that I talk about my work in a negative way."	Strongly disagree<br>Disagree<br>Agree<br>Strongly agree
"After work, I tend to need more time than in the past in order to relax and feel better."	Strongly disagree<br>Disagree<br>Agree<br>Strongly agree
"I can tolerate the pressure of my work very well."	Strongly disagree<br>Disagree<br>Agree<br>Strongly agree
"Lately, I tend to think less at work and do my job almost mechanically."	Strongly disagree<br>Disagree<br>Agree<br>Strongly agree
"I find my work to be a positive challenge."	Strongly disagree<br>Disagree<br>Agree<br>Strongly agree
"During my work, I often feel emotionally drained."	Strongly disagree<br>Disagree<br>Agree<br>Strongly agree
"Over time, one can become disconnected from this type of work."	Strongly disagree<br>Disagree<br>Agree<br>Strongly agree

"After working, I have enough energy for my leisure activities."	Strongly disagree<br>Disagree<br>Agree<br>Strongly agree
"Sometimes I feel sickened by my work tasks."	Strongly disagree<br>Disagree<br>Agree<br>Strongly agree
"After my work, I usually feel worn out and weary."	Strongly disagree<br>Disagree<br>Agree<br>Strongly agree
"This is the only type of work that I can imagine myself doing."	Strongly disagree<br>Disagree<br>Agree<br>Strongly agree
"I feel more and more engaged in my work."	Strongly disagree<br>Disagree<br>Agree<br>Strongly agree
"When I work, I usually feel energized."	Strongly disagree<br>Disagree<br>Agree<br>Strongly agree
"Usually, I can manage the amount of my work well."	Strongly disagree<br>Disagree<br>Agree<br>Strongly agree

Scoring
16-20 = very low risk;
21-25 = low risk;
26-42 =high risk
43+ = very high risk

14-item Resilience Scale (RS–14)

What is resilience?

Resilience is your ability to deal with problems, adapt to change, overcome obstacles or to resist pressure in difficult situations.

It's also the ability to react positively to those situations without having psychological or emotional conflicts.

Please tell us how much you agree or disagree with the following statements.

"I usually manage one way or another."	Completely disagree<br>Strongly disagree<br>Somewhat disagree<br>Neither agree nor disagree<br>Somewhat agree<br>Strongly agree<br>Completely agree
"I feel proud that I have accomplished things in my life."	Completely disagree<br>Strongly disagree<br>Somewhat disagree<br>Neither agree nor disagree<br>Somewhat agree<br>Strongly agree<br>Completely agree
"I usually take things in stride/calmly."	Completely disagree<br>Strongly disagree<br>Somewhat disagree<br>Neither agree nor disagree<br>Somewhat agree<br>Strongly agree<br>Completely agree
"I am friends with myself."	Completely disagree<br>Strongly disagree<br>Somewhat disagree<br>Neither agree nor disagree<br>Somewhat agree<br>Strongly agree<br>Completely agree
"I feel that I can handle many things at a time."	Completely disagree<br>Strongly disagree<br>Somewhat disagree<br>Neither agree nor disagree<br>Somewhat agree<br>Strongly agree<br>Completely agree
"I am determined."	Completely disagree<br>Strongly disagree<br>Somewhat disagree<br>Neither agree nor disagree<br>Somewhat agree<br>Strongly agree<br>Completely agree

"I can get through difficult times because I've experienced difficulty before."	Completely disagree<br>Strongly disagree<br>Somewhat disagree<br>Neither agree nor disagree<br>Somewhat agree<br>Strongly agree<br>Completely agree
"I have self-discipline."	Completely disagree<br>Strongly disagree<br>Somewhat disagree<br>Neither agree nor disagree<br>Somewhat agree<br>Strongly agree<br>Completely agree
"I keep interested in things."	Completely disagree<br>Strongly disagree<br>Somewhat disagree<br>Neither agree nor disagree<br>Somewhat agree<br>Strongly agree<br>Completely agree
"I can usually find something to laugh about."	Completely disagree<br>Strongly disagree<br>Somewhat disagree<br>Neither agree nor disagree<br>Somewhat agree<br>Strongly agree<br>Completely agree
"My belief in myself gets me through hard times."	Completely disagree<br>Strongly disagree<br>Somewhat disagree<br>Neither agree nor disagree<br>Somewhat agree<br>Strongly agree<br>Completely agree
"In an emergency, I'm someone people can generally rely on."	Completely disagree<br>Strongly disagree<br>Somewhat disagree<br>Neither agree nor disagree<br>Somewhat agree<br>Strongly agree<br>Completely agree
"My life has meaning."	Completely disagree<br>Strongly disagree<br>Somewhat disagree<br>Neither agree nor disagree<br>Somewhat agree<br>Strongly agree<br>Completely agree

"When I'm in a difficult situation, I can usually find my way out of it."

- Completely disagree
- Strongly disagree
- Somewhat disagree
- Neither agree nor disagree
- Somewhat agree
- Strongly agree
- Completely agree

Scoring

14-56	Very low (level)
57-73	Low – Moderately low (level)
74-90	Moderate – Moderately high (level)
91-98	High (level)

Thank you for answering these questions. Next we will show you your scores for each questionnaire.

UCLA Short (three-item) Loneliness Scale

What is loneliness?

On the whole, loneliness is described as an unwelcome, painful and unpleasant feeling that occurs when this is a gap, or a mismatch, between the number and quality of social relationships and connections that we have, and those we would like.
Social loneliness occurs when someone is missing a wider social network and emotional loneliness is caused when you miss an intimate relationship.

Next, please tell us how you feel about your relationships with others. Remember, when the term "others" is used, it includes colleagues, friends, neighbors, family members, or intimate partners. Check the circle that represents your response.

In general, how often do you feel that you lack companionship?

- Hardly ever
- Some of the time
- Often

In general, how often do you feel left out?

- Hardly ever
- Some of the time
- Often

In general, how often do you feel isolated from others?

- Hardly ever
- Some of the time
- Often

Scoring

3-5	Non-lonely = Low risk
6-9	Lonely = High risk

Resilience-building Behaviours

Please tell us how often you have done the following activities over the past 2 weeks:

	None in the past 2 weeks	Once or twice in the past 2 weeks	Several times per week (less than 5 times)	Daily (at least 5 times per week)
How often have you done stress management by understanding your negative thoughts and taking positive actions over the last 2 weeks?				
How often have you practiced self-reflection, mindfulness and relaxation exercises over the last 2 weeks?				
How often have you practiced Self-Care to promote your own physical, mental, and emotional health over the last 2 weeks?<br>Self-care includes<br>• Physical Self-Care like getting enough sleep and eating well<br>• Social Self-Care like spending time with family and friends<br>• Mental Self-Care like activities that mentally stimulate you<br>• Spiritual Self-Care like meditation, attending a religious service, or praying<br>• Emotional Self-Care like processing your feelings and dealing with uncomfortable emotions, like anger, anxiety, and sadness				
How often have you tried to give purpose to your professional and private life over the last 2 weeks? Giving purpose to your professional life means you like your work and look forward to doing it on most days.				
How often did you connect to others over the last 2 weeks?<br>Others include family, friends, colleagues, etc.				
