# Supplementary material for: Effectiveness of a chatbot in improving the mental wellbeing of health workers in Malawi during the COVID-19 pandemic: A randomized, controlled trial
Source: PLoS One. 2024 May 28;19(5):e0303370. doi: 10.1371/journal.pone.0303370 (PMC11132445; doi:10.1371/journal.pone.0303370)
Supplement: Attachment — Submitted filename: Plos one clinical trial review.docx [file pone.0303370.s006.docx]

**The research question posed was clear as** RCT in Malawai based on the hypothesis virtual health care assistant Chabot, Vitatalk is an reliable source of psychosocial and mental wellbeing for healthcare workers to effectively decrease work-related anxiety, depression, burnout, and loneliness (based on standard mental health scales), and to increase resilience and resilience-building behaviors.

There are primary outcome measure was done using the following survey instruments that were validated in Malawi or the Low and middle income countries.

1. The PHQ-9 has been widely used and validated in Malawi
2. Oldenburg Burnout Inventory (OLBI) has been validated and used in low- and middle-income countries (LMICs)
3. Generalized Anxiety Disorder (GAD-7) GAD-7 has been utilized effectively in Malawi and other LMICs with comparable demographics
4. UCLA Short (three-item) Loneliness Scale (UCLA Loneliness
5. 5-item Resilience-Building Behavior Scale- we did not find any publications on the validity and reliability of instruments measuring resilience building activities.
6. 14-item Resilience Scale (RS–14-) has been validated and used in LMICs

There are secondary outcome measures done using the following online questionnaires

1. Mood meter
2. Participant engagement
3. Smartphone experience
4. Current or past exposure to mental health counseling
5. Current or past chat app and website use for mental health
6. Impact of COVID-19
7. Anonymous online participant experience questionnaire
8. Focus Group Discussions

The population of interest specified was Health care workers

The sampling frame specified as 8 professional cadres eligible for participation were recruited from all public and private primary, secondary and tertiary care facilities within Blantyre and Lilongwe districts. Type of health workers eligible:• Doctors • Nurses • Medical Assistants • Clinical officers • Laboratory technicians • Physiotherapy technicians • Pharmacists • Physiotherapists.

Treatment group: Participants in the treatment group were asked to download and register with the mental health 216 chatbot app, Vitalk, and to use it daily over the 8-week study period.

Control group :Participants in the control group were provided access to a webpage in their Internet browser with links to 4 mental health resources: Mental Health Foundation in the United Kingdom]; WHO/Geneva mental well-being resources for the public; WHO/Geneva #Healthy At Home - Mental health; and the Doing What Matters in Times of Stress: An illustrated Guide.

Study design: This study, a 2-arm, parallel RCT, with a pre-treatment assessment, 8-week intervention period, mid study assessments at 4-5 weeks, and an end-of-study assessment at 9 weeks, was conducted by 2 study teams with distinct roles: a trial management team and a research team.

Participants: To be eligible, participants needed to meet the following inclusion criteria: ● Be currently employed as 1 of the 8 types of service providers listed above (a minimum 146 educational qualification of a diploma adhering to the Ministry of Health employment criteria; medical assistants with a certificate and 2 years of college completed qualified) ● Possess some degree of English language proficiency ● Own a smartphone with an Android operating system ● Have no history – past or current – of counseling or therapy for severe mental health disorders ● Not have self-reported suicidal ideation (question 9 of the PHQ-9) ● Score below “very high risk” levels for depression, or below “very high risk” of anxiety combined with “high” or “very high risk” of depression on the initial and subsequent assessments

The management team emailed excluded participants Internet resources for self-help. For those excluded on the basis of suicidal ideation or very high risk of depression and/or anxiety, the team also sent the contacts of 2 local psychologists.

Sample size: Appropriate sample size has been calculates

● Pre-post-treatment difference of 1.5 points on standard test scales ● Standard deviation of the difference = 6 ● Power 80% 11 ● Significance level 5%

This required a sample of 512, participants per arm to yield an effective post-intervention sample of **128 per arm**. Furthermore, if 20% of potential participants did not meet inclusion criteria or dropped out for other reasons, about 640 enrollees were needed initially per arm. Total enrolled were 1500 participants.

Strategy to recruit Participants: were recruited via posters placed in participating health facilities. All facilities were contacted by phone and email; in larger facilities the research team made short presentations to leadership to seek permission to recruit from their facilities and hang the posters

The strategy used to retain participants was that both study groups received weekly email and WhatsApp messages encouraging them to engage with their app or website resources. During the baseline period, the management team provided the treatment group with detailed download and sign-in instructions for the app via WhatsApp, after noticing low sign-in and receiving messages about sign-in difficulties.

Data management and analysis plan: I am not well versed with this type of analysis to comment comprehensively on it.

Results: I am not well versed with this type of analysis to comment comprehensively on it.

Discussion: It supports the results with comparison from different countries globally and regionally.

Limitations: These have been stated.

References: Most of the references are with last 10 years. Original research and reviews, RCTS, Systemic reviews, Meta- analysis, and research from global and regional studies is cited, and references for the survey instruments have been cited.
