# Supplementary material for: Effectiveness of a chatbot in improving the mental wellbeing of health workers in Malawi during the COVID-19 pandemic: A randomized, controlled trial
Source: PLoS One. 2024 May 28;19(5):e0303370. doi: 10.1371/journal.pone.0303370 (PMC11132445; doi:10.1371/journal.pone.0303370)
Supplement: Attachment — Submitted filename: Response to Reviewers.docx [file pone.0303370.s007.docx]

Dear Dr. González-Blanch and editorial reviewers,

*Thank you for these detailed and constructive reviews. We believe that all the changes have substantially improved the submission. The research team has addressed all the PLOS ON reviewer comments and questions in the revised manuscript. The revised manuscript and unedited manuscript have been uploaded, and responses to revisions are detailed below (in italics). We hope this clarifies all the comments and feedback. We would be happy to address any remaining questions.*

**Journal Requirements:**

When submitting your revision, we need you to address these additional requirements.

1. Please ensure that your manuscript meets PLOS ONE's style requirements, including those for file naming. The PLOS ONE style templates can be found at

*We reviewed the guidelines and made adjustments to the manuscript.*

2. Thank you for stating in your Funding Statement:

‘Financial support for this RCT was provided by the United States Agency for International Development under the Cooperative Agreement No. AID-OAA-A-15-00046

https://www.usaid.gov

The funders had no role in study design, data collection and analysis, decision to publish, or preparation of the manuscript.”

Please provide an amended statement that declares *all* the funding or sources of support (whether external or internal to your organization) received during this study, as detailed online in our guide for authors at http://journals.plos.org/plosone/s/submit-now. Please also include the statement “There was no additional external funding received for this study.” in your updated Funding Statement.

Please include your amended Funding Statement within your cover letter. We will change the online submission form on your behalf.

*We added the funding statement to the revised cover letter.*

3. We note that you have stated that you will provide repository information for your data at acceptance. Should your manuscript be accepted for publication, we will hold it until you provide the relevant accession numbers or DOIs necessary to access your data. If you wish to make changes to your Data Availability statement, please describe these changes in your cover letter and we will update your Data Availability statement to reflect the information you provide.

*We have updated our data availability statement in the cover letter and submitted the data as supplemental information (S2).*

**Reviewers' comments:**

Comments to the Author

1. Is the manuscript technically sound, and do the data support the conclusions?

The manuscript must describe a technically sound piece of scientific research with data that supports the conclusions. Experiments must have been conducted rigorously, with appropriate controls, replication, and sample sizes. The conclusions must be drawn appropriately based on the data presented.

Reviewer #1: Partly

Reviewer #2: Yes

Reviewer #3: Yes

Reviewer #4: Yes

*We have reviewed our manuscript and are confident that it meets the criteria as described above. We have clearly described the methodology, the primary and secondary outcomes, and based our conclusions on the findings.*

2. Has the statistical analysis been performed appropriately and rigorously?

Reviewer #1: No

Reviewer #2: Yes

Reviewer #3: Yes

Reviewer #4: I Don't Know

*We have used three commonly accepted analytic methods for this type of RCT involving mental health outcomes using validated scales: mixed-effects linear models to assess difference-in-differences estimators, effect size estimates using Cohen’s d, and reliable change in risk levels. We would side with reviewers 2 and 3 that our analysis is appropriate and rigorous.*

3. Have the authors made all data underlying the findings in their manuscript fully available?

The PLOS Data policy requires authors to make all data underlying the findings described in their manuscript fully available without restriction, with rare exception (please refer to the Data Availability Statement in the manuscript PDF file). The data should be provided as part of the manuscript or its supporting information, or deposited to a public repository. For example, in addition to summary statistics, the data points behind means, medians and variance measures should be available. If there are restrictions on publicly sharing data—e.g. participant privacy or use of data from a third party—those must be specified.

Reviewer #1: Yes

Reviewer #2: Yes

Reviewer #3: Yes

Reviewer #4: No

*A minimal anonymized data set necessary to replicate the study finding is uploaded as Supporting Information.*

4. Is the manuscript presented in an intelligible fashion and written in standard English?

PLOS ONE does not copyedit accepted manuscripts, so the language in submitted articles must be clear, correct, and unambiguous. Any typographical or grammatical errors should be corrected at revision, so please note any specific errors here.

Reviewer #1: Yes

Reviewer #2: Yes

Reviewer #3: Yes

Reviewer #4: Yes

*We copy edited the entire manuscript and made the appropriate changes as markups.*

5. Review Comments to the Author

Please use the space provided to explain your answers to the questions above. You may also include additional comments for the author, including concerns about dual publication, research ethics, or publication ethics. (Please upload your review as an attachment if it exceeds 20,000 characters)

Reviewer #1: The study is quite interesting where it investigates whether the interactive chatbot, Vitalk, is more effective than the passive use of internet resources in improving mental wellbeing and resilience outcomes of health workers in Malawi.

However, the manuscript could be further improved.

Comments

*The line numbers changed from the original due to formatting changes to comply with PLOS ONE style guidelines and additional edits. Line numbers in our responses refer to the revised manuscript* ***with all markups shown****.*

Line 115, Line 206, the description of allocation concealment is to be mentioned.

*Added language to lines 119-121 and 217-221*

Line 165, Line 168, for $10, the currency involved e,g in USD is to be clearly stated.

*Clarified on lines 171 and 174.*

Line 167, for ‘web resources At the startup’ small a for At

*Corrected on line 173, a period was missing.*

Line 195, the outcome variable, 1 or 2-tailed test used in the sample size calculation, what was the ‘difference’ refers to is to be stated.

*Clarified on lines 201-202*

Line 365, the sentence ‘days every week; and I did not work at all (coded as missing)’ not clear.

*Clarified on lines 380-381*

Information on missing data (if any) e.g. percentage, and type of missing data is to be stated.

*Thank you for pointing this out, this is important. We addressed missing data on lines 463-468, added numbers and percentages to Table 3, and added the following text. Using complete case analysis (CCA), only participants who finished base- and endline assessments were included in the calculations of DiD estimators using mixed-effects linear modeling, effect size estimation, and change in risk levels. We used CCA because missing data seem to be random as shown in the CONSORT diagram (Fig 1), which includes access to the Vitalk app or website issues, and conditionally independent of the study outcome measures. Moreover, study population characteristics of all participants with valid mental health and resilience assessments were very similar in the control and treatment groups (Fig 4). Table 3 shows the effective sample size and missing data by type of assessment. The much higher proportion of missing data for the loneliness and resilience building assessments is because participants had difficulties accessing these assessments, which were not part of the original Vitalk app but were added for this study.*

Line 381, difference-in-differences (DiD) estimator is to be defined.

*Added to lines 400-403. The DID estimator compares the differences in outcomes before and after treatment for the treatment group with any changes in the control group. It is a measure of additional change between base- and endline in the treatment group, if the value is statistically significant at a 0.05 level or smaller.*

Line 413, p ≤ 0.05 is preferable to p≤ 0.10 as indicative of statistical significance. Consideration of low P values (e.g., P<0.10) as trending toward statistical significance may be clinically relevant for improving practice, particularly in smaller studies.

*We agree and added similar language on lines 435-437.*

Line 450, to state around 29.

*Done on line 488*

Line 454, to state around 8.

*Done on line 492*

Line 458, what ‘except Age’ refers to not clear.

*Clarified as follows on lines 496: (n = 836 [481 control group, 355 treatment group] for all variables except Age with n = 828)*

Line 462, comma for figures to be replaced with dot

*We understand that there are two standards and would like to keep the comma separator. In scientific literature, including PLOS One it is common for numbers greater than 1,000 to use commas to separate groups of three digits.*

Line 462-472, figures to be presented in table.

*Done on lines 500-519, and the text was shortened accordingly to avoid repetition.*

Line 481, typo 11.4 (should be 11.04)

*Numbers were deleted from the text.*

Line 462-472, median and range values are to be omitted since mean ± sd is used.

*Done in Table 5.*

Line 554, resilience change is not the same for both groups.

*We checked the data again. Yes, the total change in resilience scores is greater in the treatment group (fig 9), but the reliable change with 13% was the same in the treatment and control groups, which is shown in Fig 8. Refers now to line 607.*

Line 594, the statement is incorrect.

*We added language about non-significant results at the 0.05 level on line 652.*

Line 599-600, Line 636 -637, Line 639-640, the data to be presented in table form.

*All data cited are presented in Table 7.*

Line 612, surgery (depression -1.53 points)

*This is addressed on line 668.*

Line 613, special care (depression -2.06)

*This is addressed on line 668.*

Table 4, median score to be omitted. Frequency to be replaced with ‘n’

*Done, now table 5*

Line 526, the sentence is unclear.

*We clarified this on lines 581-582.*

Line 543, either mean or median scores are to be reported.

*We are now reporting only mean scores.*

The percentage figures in text are to be reported at least 1 decimal point.

*We appreciate that there are different views on the number of decimals to use with percentages. We would like to stick with the NCES Statistical Standards, which states that percentages appearing in text must be rounded to whole numbers unless small differences require finer breakdowns. Using decimals in our case would suggest a level of precision and clinical significance that the self-assessments we used in our trial and our data do not justify.*

Table 6, statistical test to be denoted in the table footnote.

*Cited on line 657/658.*

Line 602, 659, symbol <= to be replaced with symbol ≤

*Done throughout the manuscript.*

Line 656, Line 659 the significant level 0.05 to be used.

*We addressed this on lines 652/709-710.*

Ensure all figures cited in the text are to be cited exactly from table(s).

*We rechecked the entire manuscript to ensure that this is the case.*

95%CI figures presented in the text could be placed in the table instead.

*Good suggestion, and we replaced SEs with 95% CIs..*

Reviewer #2: Thank you for the paper. I found the study very interesting.

The need for such as tool is extremely high in Malawi given the very limited access to psychiatric care.. Evaluation of the value of chatbots in low-resource settings and specifically for helping health worker mental health is extremely important. I am pleased to see such a trial having been conducted and even more pleased that the results show the effectiveness of such an intervention.

*Thank you for the positive feedback, it is much appreciated by all authors.*

The recruitment of over 1500 health workers and the retention rates are great achievements. The study has been well-designed, analysed and written up. The study conducted is well-designed and gathers data over an extended period (8 weeks) with mid-study data collection. A good number of participants were included.

*Thank you, this is greatly appreciated.*

Regardless of the intervention and its efficacy, the results that analyse, for example depression, anxiety and burnout, for different cadres is interesting information that may not be available otherwise.

Limitations are acknowledged.

Questions and further specific comments appear below.

It is great that the study provided funding to provide data package and transportation costs. I would hope that Vitalk could be deployed without disadvantage or bias due to cost of data packages. How much technical or other support was needed for participants to use the tool on their phones? I would hope that financial, technical and other support could be provided in the future from other sources to ensure the tool is able to be useful.

*Thank you for sharing these important points. Today’s costs of accessing the internet through mobile phones are a major barrier to using an app such as Vitalk in LMICs. However, given the rapid growth of mobile networks in low-income countries over the past decade, we anticipate that costs will come down in the foreseeable future and access will be easier without resorting to subsidies. We address some of these issues in the discussion, limitations, and conclusion sections of the revised manuscript.*

Do the authors believe the tool will continue to be useful in this COVID-normal and post-COVID period? What challenges and possible solutions are envisaged for the future maintenance, scalability, deployment and ongoing provision of Vitalk?

*We added language on lines 958-955.*

It was good to hear that there was a risk management process in terms of recruitment exclusion criteria and triggering via PHQ-9 responses. Also clarifying the chatbot does not seek to replace a human expert and help should be sought if needed.

*It is good to agree.*

I think removal of NLU is a wise move to avoid errors which may occur and increase the risk of harmful or inappropriate chatbot responses.

*An interesting observation. We will have to face the reality that NLU will rapidly become an integral part of chatbots; something that should be carefully evaluated.*

Viki is in Figure 3 not in Figure 2.

*Corrected.*

What was the motivation for including the UCLA Loneliness scale?

*Thank you for this question. Workplace loneliness was not addressed in the i-CBT or chatbot literature, but we included it based on CBT theory. A recent meta-analysis that was not available at the time of the study showed the link between loneliness and emotional wellbeing. Lines 797-799 address this issue, and we added a new citation to the article by Bryan et al.*

Why was 'I did not work at all' coded as missing?

*The questions were posed to health workers who were active in their profession for at least 12 months prior to the study. None of the work-related questions about mental wellbeing would apply to someone who did not work during the period in question.*

The sample size used significance level of .05. Why was P ≤ 0.10 used for statistical significance? This is unusual. Most commonly p<=.05 is used.

*We agree and changed the text accordingly on lines 435-437.*

Do you have any thoughts on the differences in numbers between the two groups at different study periods. The addition of percentages of the total might be useful to aid the comparisons. Why did the control group have fewer participants at midline and post-endline and more at baseline and endline?

*We added percentages to Table 2 and added an explanation related to the passive and interactive nature in the control and treatment groups respectively on lines 459-462.*

Only participant who completed -->

Only participants who completed

*Corrected.*

I am surprised that almost all participants did not report that the pandemic had disrupted their work.

*Anecdotal evidence suggests that health services operated during the pandemic in Malawi.*

p. 23 Why wasn't mood status for the control group measured?

*Lines 340/341: The mood meter was part of the intervention to raise self-awareness, and only available to the treatment group.*

Control group is lower on baselines, what is the possible impact on interpretation of other results of these sig diffs. This means it would have been harder to show a difference following treatment.

So there is a significant difference for control as well, but no significant difference between groups at endline. This does not support that the intervention was useful.

Resilience is the exception.

*Based on the summary statistics, such a conclusion would seem plausible, but a comparison of group means does not tell the full story as it does not consider the change brought about by the treatment, the Vitalk app, compared to any change in the control group. The difference-in-differences method does take the change in the treatment and control groups into account and showed that the treatment did result in greater change. In addition, the effect size estimates showed a greater effect in the treatment than in the control group. Lastly, the proportion of participants that changed risk levels in a positive direction (lower for anxiety, depression, and burnout, higher for resilience and resilience building) was higher in the treatment group. The proportion of participants that changed risk levels in a negative direction (higher for anxiety, depression, and burnout, lower for resilience and resilience building) was higher in the control group. All these metrics together show that Vitalk is effective in improving mental health and resilience. We edited lines 532-538 accordingly.*

p.24 deceases in depression -->

decreases in depression

*Corrected*

p. 25 printing /formatting error at line 504.

*We could not find this in the downloaded manuscript. We will have to look for it in the next submission.*

Only DiD estimators seem to show statistically significant differences. Why is this the case, when t-tests do not show significant differences?

*T-tests at endline only compare the groups at a single point in time (post-intervention). However, the outcome variable is influenced by time trends based on the treatment in the treatment and control groups. DiD estimators compare the changes in the outcome variable before and after the intervention for both the treatment and control groups. DiD estimators also attempt to control for unobserved differences, which can still exist in an RCT, by comparing the change in the outcome variable for the treatment group relative to the change in the outcome variable for the control group after the intervention.*

p.30 "None of the 95% CI contain zero, indicating that both study groups experienced a positive effect"

How do you account for this? Were the resources potentially useful, though they were not accessed very much. Or is being alerted to how you are feeling, via doing the baseline enough to help you feel better or feel that someone else cares to ask?

*Both are likely explanations of a positive effect in both study groups. We included similar language and added test bias on lines 590-592.*

p.31 "very good (greater and 0.80)"

--> "very good (greater than 0.80)"

*Corrected*

p. 31 "did not impart whether these were of clinical relevance."

impart??

*Changed to “inform”.*

Reviewer #3: This is an interesting paper reporting on the first RCT of a mental health app for healthcare workers during the COVID19 pandemic in Southern Africa combining multiple mental wellbeing outcomes, and measuring resilience and resilience-building activities. It is a well formulated and reported study which is of relevance to the mental health literature relating to the COVID-19 pandemic.

*Thank you for your supportive comment. It is much appreciated by all authors.*

Reviewer #4: The writing is clear and straightforward. The author presents the material in an organized fashion, and the figures are well-chosen. However, I have some comments.

1. The University Research Co. (URC) Institutional Review Board and the University of Malawi Research Ethics Committee (UNIMAREC) approved the study protocol – Please kindly provide the protocol number given by ethics committee.

*Added on line 164-166.*

2. Trial was registered retrospectively with the ISRCTN registry-Please kindly provide this number.

*Added on line 167.*

3. How was missing data handled?

*We addressed this on lines 453-468 providing a rationale for using a complete case analysis (CCA) approach and quantified missing data in Table 3.*

4. Strategies used to optimize the response rate were not mentioned.

*They are mentioned on lines 170-181.*

5. The method of questionnaire administration was not specified.

*Good catch. We added the information on lines 122 (demographic information), 329 (mental health assessments), and 382-384 (participant experiences).*

6. There was no additional details regarding an incentive for questionnaire completion provided?

*We did not provide any direct incentives to encourage completion of online questionnaires or mental health assessments to avoid undue influence. The mobile data allowance was provided unconditionally to all study participants, which we described in the manuscript.*

7. Response rate was not defined and also it was not reported. What was done with incomplete surveys if any?

*Information about retention rates can be found on lines 765-778. We added the definition. Incomplete surveys were not included in the analysis, because participants were often trying out the mental health assessments before completing them. This is based on feedback during the final in-person study workshops. We therefore considered incomplete surveys invalid.*

8. Demographic data of the survey respondents was not clear. Though 8 categories of health care professionals was mentioned yet the figure only shows nurses.

*To show that control and treatment groups were balanced post randomization, the graphic only compares the category with the largest proportion for each demographic variable for the control and treatment groups. We did not show a more detailed breakout as that would add another page to the manuscript. Some details are part of the OLS analysis in Table 7.*

9. Were the questionnaires pilot tested?

*Yes, all mental health and resilience questions were pilot tested by Vitalk and members of the research team. We added this language on lines 276/277.*

10. How can technical difficulties minimized for others who plan similar studies as there was a high attrition rate in the Treatment group due to this?

*Good question. There were factors in the control of the study management team and others that were outside their control. Meticulous planning of the app rollout is critical, which includes ensuring local access to an app that was developed for a different region (Brazil) and not underestimating the amount of handholding necessary to install the app on smartphones for a study population that is not a frequent used of mobile apps because of costs. Our study management team spent considerable effort supporting users online, through WhatsApp groups, and individual email support. Plan for sufficient lead time to add elements to a life app, 2 additional assessments in our case and user identification, to ensure smooth operation. Factors to take into account but are beyond the influence of the study included intermittent internet access, which affected both study groups equally and limited smartphone app use experience which affected the treatment group more than the control group. We added text on lines 944-957.*

11. All the questionnaires used should be provided as appendices.

*Yes, we included those as supplemental information (S1).*

6. PLOS authors have the option to publish the peer review history of their article (what does this mean?). If published, this will include your full peer review and any attached files.

If you choose “no”, your identity will remain anonymous but your review may still be made public.

Do you want your identity to be public for this peer review? For information about this choice, including consent withdrawal, please see our Privacy Policy.

Reviewer #1: No

Reviewer #2: No

Reviewer #3: Yes: Dr Sarah Markham

Reviewer #4: Yes: Khizra Sultana

[NOTE: If reviewer comments were submitted as an attachment file, they will be attached to this email and accessible via the submission site. Please log into your account, locate the manuscript record, and check for the action link "View Attachments". If this link does not appear, there are no attachment files.]

*We reviewed the attached file of reviewer comments. The were no questions related to manuscript content.*

While revising your submission, please upload your figure files to the Preflight Analysis and Conversion Engine (PACE) digital diagnostic tool, https://pacev2.apexcovantage.com/. PACE helps ensure that figures meet PLOS requirements. To use PACE, you must first register as a user. Registration is free. Then, login and navigate to the UPLOAD tab, where you will find detailed instructions on how to use the tool. If you encounter any issues or have any questions when using PACE, please email PLOS at figures@plos.org. Please note that Supporting Information files do not need this step.

In compliance with data protection regulations, you may request that we remove your personal registration details at any time. (Remove my information/details). Please contact the publication office if you have any questions.
